# PV-WTConv: a parallel network for abnormal white blood cell classification based on wavelet transform convolution

**DOI:** 10.3389/fmed.2026.1947482

**Published:** 2026-10-28

**Authors:** Xiangsuo Fan, Xipeng Song, Borui Sun, Zhenxing Yao, Jie Meng

**Affiliations:** 1 School of Automation, Guangxi University of Science and Technology, Liuzhou, Guangxi, China; 2 Medical Science Laboratory, Liuzhou Worker’s Hospital, Liuzhou, Guangxi, China

**Keywords:** abnormal white blood cells, deep learning, parallel network, wavelet transform convolution, white blood cell classification

## Abstract

In the field of cell detection research, the detection of abnormal white blood cells is not only central to the early diagnosis of malignant haematological diseases such as leukaemia and myelodysplastic syndromes, but also serves as a key indicator for assessing infectious diseases, immunodeficiencies and the efficacy of chemotherapy. The use of computer-aided techniques to accurately classify white blood cells in microscopic images can assist clinicians in diagnosing and detecting various blood-related diseases more promptly and accurately. However, the accurate identification and classification of white blood cells remains challenging due to the blurred boundaries between abnormal white blood cells and normal white blood cells—or even those exhibiting physiological variations—the lack of uniform and fixed morphological criteria for different types of abnormal cells (such as leukaemic cells and hyperplastic cells), and interference from factors such as the complexity of cell imaging and staining techniques. Therefore, this paper proposes a parallel classification network for abnormal white blood cells based on wavelet transform convolution, designed to enhance the accuracy of recognising and classifying different categories of abnormal white blood cells. This study utilised the AWBC-LZWH dataset of abnormal white blood cell images, provided by the Department of Clinical Laboratory Medicine at Liuzhou Workers’ Hospital, for the main training, validation and testing phases, and conducted supplementary experiments using the PBC_dataset of peripheral blood cell images, which was collected and publicly released by the Hospital Clínic de Barcelona. PV-WTConv achieved an accuracy of 94.4% on AWBC-LZWH, compared with 56.9% for the convolutional baseline and 91.4% for ResNet50, corresponding to absolute differences of 37.5 and 3.0 percentage points, respectively. The results of this study indicate that PV-WTConv demonstrates good classification performance on the evaluated white blood cell image datasets; however, its clinical diagnostic value still requires further validation through prospective, multicentre studies.

## 1 Introduction

As a core component of the human immune system and the “sentinels of health” in the bloodstream (1), even subtle changes in the number, proportion and morphology of white blood cells often serve as the earliest and most direct indicators of the onset and progression of disease. This study focuses on six categories of white blood cells: abnormal promyelocytes in acute promyelocytic leukaemia (APL; FAB M3), suspected neoplastic abnormal lymphocytes, myeloblasts (AML), normal white blood cells, lymphoblasts (ALL) and reactive lymphocytes. Accurate differentiation between these categories facilitates the analysis of white blood cell morphology and associated changes, and provides supplementary information for subsequent clinical assessment. Normal white blood cells form the cornerstone of defence against infection and the clearance of foreign substances; however, abnormalities in their development, differentiation or function can lead to the emergence of Abnormal promyelocytes in acute promyelocytic leukemia (APL; FAB M3), Myeloblasts (AML) and Lymphoblasts (ALL). Conversely, when these cells are stimulated to produce excessive or abnormal responses, conditions such as Abnormal lymphocytes (suspected neoplastic) and Reactive lymphocytes may arise, signalling an imbalance in the immune system or even the onset of malignant diseases. Accurately distinguishing between these cell types—particularly identifying Lymphoblasts (ALL) cells intermingled with Normal leukocytes or abnormal cells with clear diagnostic significance (such as the M3 subtype)—is akin to pinpointing the weak points or points of betrayal in the immune defence system. This not only directly determines the precise subtyping of acute leukaemias (such as APL, AML and ALL) and the formulation of emergency treatment plans, but is also crucial for distinguishing between malignant lymphoproliferation and benign reactive hyperplasia. It plays an irreplaceable and decisive role in avoiding misdiagnosis and missed diagnoses, realising personalised treatment, and assessing disease risk and prognosis. Consequently, the precise classification of these six cell types essentially provides a window into the body’s vital “immune cell pool”, enabling cutting-edge insights into and precise interventions for the health and disease states of the haematological system; it is a crucial step in safeguarding the defences of life and health (2). Traditional manual microscopic identification methods face insurmountable inherent limitations when confronted with such complex, intricate and clinically significant classification tasks (3). Manual interpretation relies heavily on the experience and subjective judgement of laboratory technicians, inevitably introducing significant inter-observer and intra-observer variability, thereby compromising the consistency and objectivity of diagnostic results (4). Furthermore, due to the high degree of morphological overlap or the extremely subtle and atypical nature of these features, the human eye has limited resolution and stability when identifying these categories. This makes it highly prone to missing or misidentifying critical abnormal cells, posing a significant risk in critical cases—such as those involving M3 leukaemia—where urgent and precise classification is required to initiate specific treatment. In addition, manual screening of vast quantities of cell images is inefficient and time-consuming, making it difficult to meet clinical demands for high-throughput, rapid reporting (4). Computer-aided diagnosis (CAD) technology and machine learning (ML) methods are widely applied in the field of medical image analysis, particularly in addressing technical bottlenecks in white blood cell (WBC) detection and classification (5). Numerous studies have focused on the intelligent analysis of peripheral blood smear images, utilising algorithms to achieve automatic identification, subtyping and quantitative analysis of white blood cells. However, the performance of existing traditional machine learning methods is often constrained by the validity and generalisation ability of manually designed features, making it difficult to fully capture the complex, subtle and variable morphological and textural differences in white blood cell images; traditional data processing methods also exhibit significant limitations when handling large-scale, complex data, prompting researchers to develop more intelligent data mining techniques (6). Consequently, this paper proposes the PV-WTConv classification framework based on CNNs. In the initial feature extraction stage, this framework employs a parallel configuration comprising a top-hat transform, wavelet convolution (WT-Conv) and traditional convolution (Conv). The top-hat transform is used to enhance bright regions and morphological details within cells, whilst wavelet convolution extracts high- and low-frequency features from a multi-scale, multi-frequency perspective; conventional convolution, meanwhile, focuses on local textural information, thereby alleviating the challenges posed by the intricate details of abnormal white blood cells and the subtle morphological differences between categories. Subsequently, a Vision Transformer (ViT) module is integrated; through its self-attention mechanism, the ViT module is used to model long-range relationships between different regions of an image, whilst ParNetAttention is used to process the resulting features; together, they form the feature learning process for the classification of abnormal white blood cells. The main contributions of this paper are as follows. We propose a parallel classification network for abnormal white blood cells, PV-WTConv, based on wavelet transform convolution, designed to improve the classification accuracy and robustness of microscopic images of abnormal white blood cells. A parallel feature extraction module comprising a top-hat transform, wavelet convolution (WT-Conv) and conventional convolution (Conv) has been constructed. This module combines morphological information, multi-scale frequency-domain information and local texture information to characterise the complex morphology of abnormal white blood cells and the subtle differences between categories. A Vision Transformer module was introduced to model long-range dependencies between different image regions via a self-attention mechanism, thereby addressing the limitations of the local receptive field in traditional convolutional networks and enhancing global semantic representation capabilities. Concurrently, the ParNetAttention mechanism was incorporated to further filter and enhance key features, improving the model’s ability to distinguish subtle morphological differences whilst balancing local details with global correlations. On AWBC-LZWH, PV-WTConv achieved an accuracy of 94.4%, exceeding the convolutional baseline and ResNet50 by 37.5 and 3.0 percentage points, respectively, under the reported experimental settings. These results illustrate the classification performance of the various models under the experimental conditions of this study. The remainder of this paper is organised as follows: Section 2 introduces relevant work on white blood cell image analysis; Section 3 details the proposed PV-WTConv network architecture and its constituent modules; Section 4 presents the experimental setup, evaluation metrics, and results of comparative and ablation experiments; Section 5 discusses the results and limitations of the study.

## 2 Related work

In recent years, medical image object detection has emerged as a significant research area in medical artificial intelligence. White blood cell image analysis presents numerous challenges, including the high heterogeneity of cell morphology, variations in staining, and stringent accuracy requirements for clinical diagnostics (7). Classifiers such as Bayesian, Random Forest, KNN, and SVM are commonly used for white blood cell detection and classification tasks in medical image analysis. Alternatively, deep learning enables end-to-end learning, allowing direct category learning from raw images.

### 2.1 Traditional classification methods

Research on white blood cell recognition technology dates back to the 1960s, when researchers primarily employed basic image segmentation algorithms combined with traditional classification methods to achieve cell detection. Entering the 21st century, with the advent of advanced classifiers such as Support Vector Machines (SVM), researchers began applying multi-algorithm fusion strategies to white blood cell image analysis, with reported improvements in recognition accuracy. In 2014, (8) used a Gaussian kernel-based SVM algorithm to classify 267 white blood cells into five major categories, correctly identifying 245 out of 267 cells (92% accuracy). In 2015, (9) integrated thresholding, morphological operations, and ellipse fitting during segmentation. They extracted desired features from segmented nuclear and cytoplasmic regions, then classified white blood cells using selected prominent features via linear and naive Bayes classifiers. The overall classification accuracies for the linear and naive Bayes models during classification reached approximately 98% and 94%, respectively. In 2016, (10) proposed using Wiener filtering and Curvelet transformation for image enhancement and noise reduction to avoid false edges. Subsequently, a combination of entropy filtering, thresholding, and mathematical morphology was employed for image segmentation and boundary detection. A backpropagation neural network was then used to classify white blood cells into their respective subtypes. Accuracy rates of 100%, 96.15%, 92.30%, 92.30%, and 96.15% were achieved for basophils, eosinophils, monocytes, lymphocytes, and neutrophils, respectively. In 2016, (11) proposed a dual-threshold approach based on the strategic combination of RGB and HSV color spaces for white blood cell segmentation. This method improved upon traditional single-threshold techniques and employed a golden ratio search algorithm to determine optimal thresholds. In 2018, (12) proposed a method that first extracts overall foreground regions from cell images using K-means clustering, then generates white blood cell regions based on concavity analysis through cell division contact. The second module trained a Support Vector Machine (SVM) classifier using the segmentation results as labels. This trained SVM classifier then classified each pixel in the image, yielding segmentation results that were evaluated on the study dataset. In 2018, (13) proposed a computer-aided system (CAS) based on color band threshold segmentation. By combining color space correction ( L×a×b ) and RGB, CMYK, and HSV color analysis with Otsu threshold segmentation, it effectively extracted WBC regions. Morphological filtering and circular Hop transform (CHT) were then applied to enhance counting accuracy. The accuracy rate reached 95.65%.

### 2.2 Deep learning classification methods

Although traditional machine learning methods have achieved some success in detecting abnormal white blood cells, their performance is often constrained by the effectiveness and generalization capabilities of manually designed features. The feature engineering process is not only cumbersome and time-consuming but also highly dependent on domain experts’ prior knowledge (14). It struggles to fully capture the complex, subtle, and variable morphological and textural differences in white blood cell images, particularly when handling challenging scenarios such as staining variations, cell overlap, and morphological variability (15). In recent years, deep learning technologies, particularly deep convolutional neural networks (CNNs), have achieved revolutionary progress in medical image analysis through their powerful end-to-end feature learning capabilities, opening new avenues for abnormal white blood cell classification. In 2017, (16) classified WBCs using CNNs and 2000 images. Comparing their results with classical methods, the authors achieved an 88.5% success rate. In 2018, (17) proposed integrating CNNs with RNNs, introducing the CNN-RNN framework. This approach deepens understanding of image content, learns structural features, and initiates end-to-end training for big data in medical image analysis. The proposed network model demonstrated enhanced accuracy in blood cell image classification. In 2018, (18) introduced a technique called transfer learning, which involves modifying pre-trained CNNs to classify different types of objects. The cited study reported reduced training time with transfer learning and an accuracy of 96.63%. In 2019, (19) employed SSD and YOLOv3 models for automated white blood cell detection, achieving 11-class peripheral white blood cell detection with accuracies of 93% and 90%. In 2020, (20) proposed a novel convolutional neural network (CNN) model. This approach combines the concept of merging features from the first and last convolutional layers while propagating input images through convolutional layers. Additionally, a dropout layer was employed to prevent overfitting. This method achieved an average accuracy of 98.61% and demonstrated strong performance on public datasets. In 2023, (21) presented a WBC classification approach based on an enhanced U-Net architecture and support vector machines (SVM). An enhanced U-Net architecture was developed for WBC segmentation, followed by feature extraction and classification using radial basis function support vector machines (RBF-SVM). The proposed U-Net-SVM achieved recognition rates of 99.45%, 98.62%, and 98.81% for WBCs in the Raabin-WBC, LISC, and BCCD datasets, respectively. In 2023, (22) proposed a network based on W-Net (a CNN-based WBC classification method) to perform white blood cell segmentation. Subsequently, a deep learning framework built on GhostNet was employed to retrieve salient feature maps. These were then classified using ResNeXt and WHO-based methods. Additionally, data augmentation via Deep Convolutional Generative Adversarial Networks (DCGAN) was utilized to expand the dataset. Ultimately, high accuracy levels of 99.16%, 99.24%, and 98.61% were achieved across three datasets. In 2024, (23) proposed a deep learning model combining a deep CNN with image processing techniques to segment and classify white blood cells into four distinct types. The proposed model was independently evaluated on each of the four white blood cell image types and reported precision, sensitivity, accuracy and F1-score results. In 2024, (24) proposed leveraging Deep Convolutional Generative Adversarial Networks (DCGANs) to overcome limitations imposed by limited training data, employing a dual attention mechanism to enhance accuracy, efficiency, and generalization. The proposed model achieved high accuracy on peripheral blood cell (PBC), leukocyte image segmentation and classification (LISC), and Raabin-WBC benchmark datasets. In 2025, (25) introduced a robust two-stage CNN framework integrating YOLOv8 for precise segmentation and MobileNetV3 for efficient classification. First, YOLOv8m-seg segments white blood cells, extracting ROIs for subsequent analysis. Subsequently, features from segmented ROIs train MobileNetV3 to classify white blood cells. This framework demonstrates strong performance across multiple datasets. In 2025, (26) introduced a dual-attention mechanism that integrates channel and spatial localization features from images, with the aim of enhancing feature representation for white blood cell classification. Building upon this, they proposed a Morphological Feature Extractor (MFE) comprising a Morphological Attribute Predictor (MAP) and a Morphological Attribute Encoder (MAE) to extract morphological features of white blood cells. To enhance MAE performance, deep supervised learning (DSL) and semi-supervised learning (SSL) training strategies were implemented. Relevant research over the past two years has further advanced deep learning methods for cell images and medical imaging: in 2024, (27) proposed a dual-branch feature fusion ensemble framework based on EfficientNetB7 and MobileNetV3Large for the diagnosis of acute leukaemia smear images; in 2025, (28) proposed a CNN diagnostic software for malaria red blood cell images and compared it with VGG19 and EfficientNetB3; in 2024, (29) combined CNN feature extraction with Ridge feature selection and the maximum information coefficient for blood cell classification; in 2026, (30) combined a VGG19 encoder with a U-Net decoder for MRI tumour segmentation, validating the effectiveness of hybrid deep architectures in medical imaging tasks; furthermore, in 2024, (31) utilised random search optimisation to compare various machine learning models, demonstrating the importance of hyperparameter optimisation and standardised evaluation metrics for analysing model performance. Consequently, these studies provide new insights for the design and experimental evaluation of the PV-WTConv framework in this paper, covering aspects such as feature fusion, transfer learning, feature selection, hybrid network architectures and the standardisation of evaluation metrics. A detailed description is provided in Table 1. Therefore, this paper proposes the CNN-based PV-WTConv classification framework.

**Table 1:** Advantages and limitations of relevant white blood cell image analysis methods.

Method category	Representative methods	Advantages	Limitations
Traditional image processing and machine learning methods	Threshold segmentation, morphological operations, SVM, Bayesian classifiers, etc.	Methods have a clear structure and are highly interpretable, and are effective to a certain extent on small datasets.	Reliance on manually extracted features and segmentation quality; limited adaptability to variations in staining, cell overlap and morphological variations.
CNN-based classification methods	CNNs, enhanced CNNs, transfer learning models, etc.	They are capable of automatically learning image features, reducing the need for manual feature design, and supporting end-to-end learning for classification.	They rely heavily on local features, whilst still falling short in representing global spatial relationships and subtle morphological differences.
Combined segmentation and classification methods	U-Net–SVM, YOLOv8–MobileNetV3, etc.	By first localising or segmenting cellular regions before classification, background interference can be reduced.	The overall workflow is relatively complex, and segmentation errors may affect subsequent classification results.
Attention and feature fusion methods	Dual Attention Network, DAFFNet, Feature Fusion Network, etc.	These enhance the representation of features in key regions and channels, improving the model’s ability to focus on important information.	Model architectures are typically more complex, placing higher demands on the volume of training data and computational resources.
Data augmentation and generative methods	DCGAN, data augmentation, etc.	These can mitigate the issue of limited training samples and improve the model’s generalisation ability.	The quality of generated samples affects the effectiveness of model training; it is still necessary to ensure that the data distribution aligns with that of real samples.

## 3 Materials and methods

### 3.1 Dataset

The AWBC-LZWH dataset comprises a total of 5,919 white blood cell microscopic images from 480 patients. Of these, “Abnormal promyelocytes in acute promyelocytic leukaemia (APL; FAB M3)” comprises 50 patients, “Abnormal lymphocytes (suspected neoplastic)” comprises 20 patients, “Myeloblasts (AML)” comprises 200 patients, “Normal leukocytes” comprises 70 patients, “Lymphoblasts (ALL)” comprises 80 patients, and “Reactive lymphocytes” comprises 60 patients. The patients and their corresponding images across the six categories are mutually exclusive; each patient is assigned to only one cell category, and there are no instances of the same patient or the same image appearing repeatedly in different categories. The training, validation and test sets of the AWBC-LZWH dataset are organised using stratified patient-level partitioning, rather than randomly distributing images from the same patient across different subsets. In accordance with an 8:1:1 ratio, the 480 patients were divided into 384 training patients, 48 validation patients and 48 test patients. Specifically, the numbers of patients in the training, validation and test sets for the APL category are 40, 5 and 5 respectively; for Abnormal lymphocytes (suspected neoplastic), 16, 2 and 2; for AML, 160, 20 and 20; for Normal leukocytes, 56, 7 and 7; for ALL, 64, 8 and 8; and for Reactive lymphocytes, 48, 6 and 6. All cell images from the same patient were assigned in their entirety to a single data subset; consequently, there is no overlap between the sets of patient IDs in the training, validation and test sets, thereby ensuring that the three data subsets are mutually independent at the patient level. As the number of cell images contributed by different patients is not entirely consistent, once the patient-level partitioning is complete, the number of images in each of the three subsets is calculated based on the actual number of images contributed by each patient. Ultimately, the training set, validation set and test set contain 4,735, 592 and 592 images respectively. The ratio of 8:1:1 here primarily refers to the patient-level partitioning ratio, whilst the figures 4,735, 592 and 592 represent the actual number of images contained in each subset following patient grouping; this does not imply a random division of all images at the image level. All subsequent model training, validation, selection of optimal weights and final testing are carried out based on the aforementioned fixed and mutually independent patient-level data subsets, as shown in Figure 1. Specifically, it includes: 1,000 images of Abnormal promyelocytes in acute promyelocytic leukemia (APL; FAB M3), 918 images of Abnormal lymphocytes (suspected neoplastic), 1,000 images of Myeloblasts (AML), 1,000 images of Normal leukocytes, 1,000 images of Lymphoblasts (ALL), and 1,001 images of Reactive lymphocytes.

**Figure 1:**
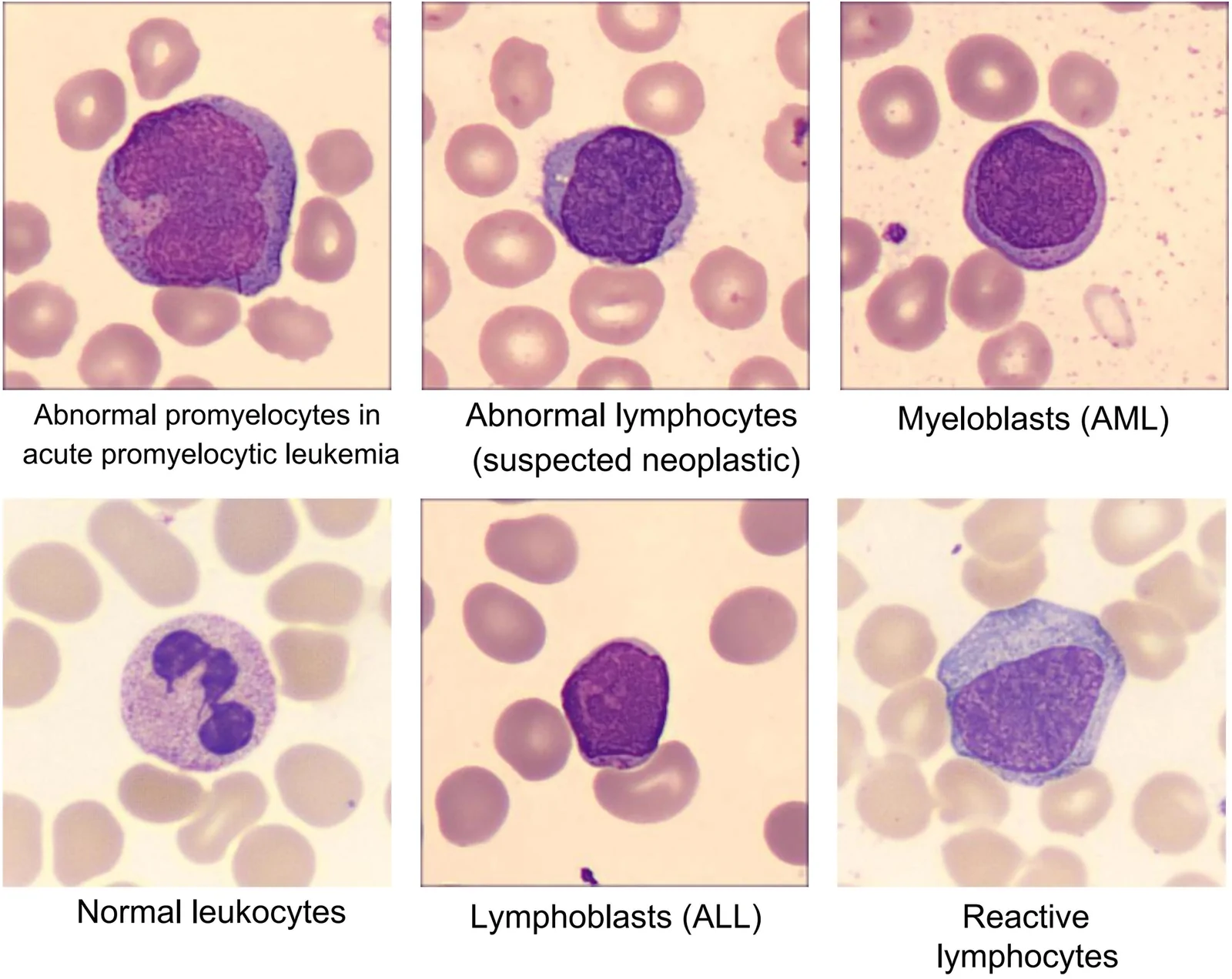
Diagram of six types of cells.

All images originate from the CellaVision-DI-60 system. The dataset consists of 360×367 pixel images derived from Wright-Giemsa-stained peripheral blood smears. These images were captured using a digital camera connected to a microscope equipped with a 100× achromatic objective lens, stored in JPG format. The annotation and review of cell types in the AWBC-LZWH dataset were carried out independently by two clinicians with extensive experience in cytomorphology. Both annotators hold practising medical licences and hold professional technical titles of associate senior level or above. They have been engaged in cytomorphology-related work for over 20 years, possess a solid theoretical foundation in haematological cytomorphology and extensive clinical experience in slide review, are capable of identifying different types of abnormal cell morphology, and are familiar with the characteristic cytomorphological manifestations associated with haematological disorders. Prior to annotation, the two annotators standardised their approaches regarding basic operational procedures, morphological interpretation logic, reporting conventions and quality control standards, in order to minimise subjective variations arising from differences in their experience of slide review. Cell categories were primarily determined based on morphological features observed in Wright–Giemsa-stained peripheral blood smears, including cell size and outline, nucleocytoplasmic ratio, nuclear morphology, chromatin features, nucleoli, cytoplasmic staining characteristics and cytoplasmic granules, and annotation was completed in accordance with predefined classification criteria. All images were reviewed using a double-blind, cross-checking method to ensure that each image underwent independent assessment by at least two annotators. For images where discrepancies arose, the source of the disagreement was first identified by verifying image quality, category entry, and other fundamental errors resulting from non-specialist judgements; subsequently, the two annotators conducted independent re-evaluations based on uniform morphological assessment criteria, using objective morphological evidence to minimise the impact of differences in experience in slide interpretation; if consensus could not be reached after re-evaluation, a senior cytomorphology expert from the department was invited to act as a third party for independent interpretation, and the final category label was determined based on the majority consensus. During the construction of the dataset for this study, quantitative inter-observer agreement metrics such as Cohen’s κ were not calculated; therefore, annotation consistency was primarily ensured through quality control measures comprising independent double-blind review by two experts and a third-party arbitration process for disputed cases. The AWBC-LZWH dataset was provided by Liuzhou Workers’ Hospital, and all data used were anonymized without personal identifiable information. Please refer to the “Data Availability Statement” for details regarding the scope of public access to this dataset and how to obtain the complete dataset. Furthermore, this study utilised a publicly available dataset of peripheral blood cell images (PBC_dataset) collected by Hospital Clínic de Barcelona to conduct supplementary experiments, with a view to further evaluating the generalisation capability of the proposed method. The data used in this study include the AWBC-LZWH dataset provided by Liuzhou Workers’ Hospital and the publicly available PBC_dataset. This study utilises the PBC_dataset—a dataset of peripheral blood cell images collected and publicly released by the Hospital Clínic de Barcelona—to further evaluate the suitability of the proposed network architecture for the independent eight-class peripheral blood cell classification task. The dataset comprises 17,092 images of normal peripheral blood cells, which were acquired by the hospital’s central laboratory using a CellaVision DM96 analyser. The dataset is divided into the following eight groups: neutrophils, eosinophils, basophils, lymphocytes, monocytes, immature granulocytes (promyelocytes, myelocytes and metamyelocytes), erythroblasts and platelets. The images are 360 × 363 pixels in size, in JPG format, and have been annotated by clinical pathologists. These images were acquired from individuals free from infection, haematological or neoplastic diseases, and who were not receiving any medication at the time of blood collection.

### 3.2 PV-WTConv

This section details the proposed PV-WTConv architecture, as shown in Figure 2. The model employs a parallel structure to sequentially advance feature extraction and classification tasks. During the initial feature extraction phase, modules comprising the Top Hat transformation, wavelet convolution (WT-Conv), and traditional convolution (Conv) operate in parallel. The Top Hat transformation precisely captures detailed information in bright regions of the image, while wavelet convolution extracts features at different scales, effectively balancing local and global information, while traditional convolution focuses on local feature extraction. These three components work in parallel to comprehensively extract effective features from the input image across complementary feature domains and multiple scales, adapting to the feature diversity of complex images like abnormal white blood cells. Subsequently, central difference convolution (CDC) and max-pooling are employed to concurrently optimize the preliminarily extracted features, filtering key information while reducing dimensionality. This iterative process of feature extraction, optimization, and fusion progressively refines and integrates features, enhancing their discriminative power for subsequent classification tasks. Finally, the Visual Transformer (VIT) is introduced to concurrently capture long-range dependencies between features, uncovering global correlations. The ParNetAttention mechanism further enhances focus on critical features. This design combines local features with global relationships, aiming to provide a complementary feature representation for white blood cell image classification. Its key architectural features include: the parallel extraction of morphological, frequency-domain and local texture information; the gradual integration of features from different stages; and the use of ViT and ParNetAttention for further processing of the fused features. The impact of this design on classification performance is evaluated through subsequent comparative and ablation experiments. To provide a clearer and more complete description of the proposed method, the overall algorithm of PV-WTConv is summarised as follows. Given an input white blood cell image, the top-hat transform, wavelet convolution (WT-Conv), and conventional convolution (Conv) are first employed in parallel to extract complementary morphological, multi-scale frequency-domain, and local texture features. The extracted features are then progressively refined and fused through central difference convolution (CDC) and max-pooling operations, followed by the Vision Transformer (ViT) to model long-range dependencies and global feature correlations. Finally, ParNetAttention further enhances the discriminative key features, and the resulting feature representation is used to obtain the final white blood cell classification result. To further clarify the implementation of PV-WTConv, this study provides a supplementary explanation of the specific interconnections between the various modules. The input is a three-channel image of 224 × 224 pixels. In the wavelet feature extraction path, the outputs of the first two WT-Conv modules undergo 1 × 1 convolutions for channel mapping, followed by downsampling via max-pooling with a kernel size and stride of 2, mapping the number of channels sequentially from 3 to 64, and then from 64 to 128. The morphological path first performs a Top-Hat operation on the input and adds it element-wise to the output of the first WT-Conv module; subsequently, the fused features from the previous stage undergo CDC, max-pooling and Top-Hat processing in sequence, before being added element-wise to the corresponding wavelet features from that stage. The final fused features are fed into the ViT module, whose output is then passed to the ParNetAttention and the final classification module. Consequently, feature fusion at each stage employs element-wise addition rather than concatenation along the channel dimension. The complete workflow of the proposed PV-WTConv algorithm is illustrated in Figure 2.

**Figure 2:**
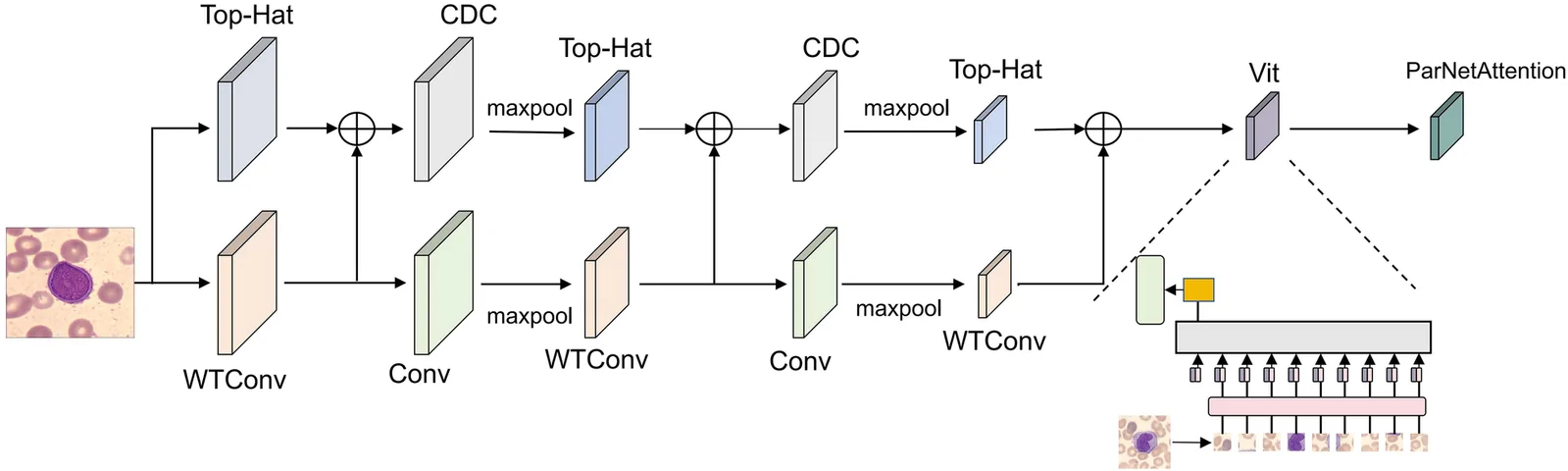
PV-WTConv structure diagrame.

#### 3.2.1 WT-Conv

Due to the limited feature extraction capabilities of conventional convolutional layers, they tend to filter both targets and noise together when processing mixed target-noise data, preventing the network from capturing critical features. However, excessive convolutional layers for feature extraction impose additional computational overhead. Therefore, this subsection introduces the Wavelet Transform Convolutional Network (WT-Conv). Input features undergo wavelet decomposition into frequency-domain components, which are then convolved to capture high-frequency and low-frequency information. High-frequency information typically represents rapidly changing pixel regions in images, including details, edges, and texture features. Low-frequency information generally represents areas with gradual pixel changes, encompassing overall color, lighting, and large-scale regional features. After extracting high- and low-frequency information, an inverse transform is applied to return the data to the spatial domain. Consequently, WT-Conv provides multi-frequency representations for subsequent feature extraction. In Figure 3, WT denotes the wavelet transform, which decomposes an image X of size W×H into four subsets: $X_{ll}$ (low-frequency), $X_{hh}$ (high-frequency), $X_{lh}$ (horizontal), and $X_{hl}$ (vertical). This transform process extracts information at different scales; the low-frequency component can be recursively processed through cascaded wavelet decomposition, as shown in Equations 1, 2.

$$X_{ll}^{i},X_{hh}^{i},X_{lh}^{i},X_{hl}^{i}=\mathrm{WT}\left(X_{ll}^{i-1}\right)$$ (1)

Y=IWT(Conv(W,WT(X))) (2)

Here, i denotes the current decomposition level. By recursively applying the wavelet transform to $X_{ll}$, a series of wavelet transforms is generated that effectively captures the low-frequency and high-frequency components of the input image, facilitating detailed feature extraction.

**Figure 3:**
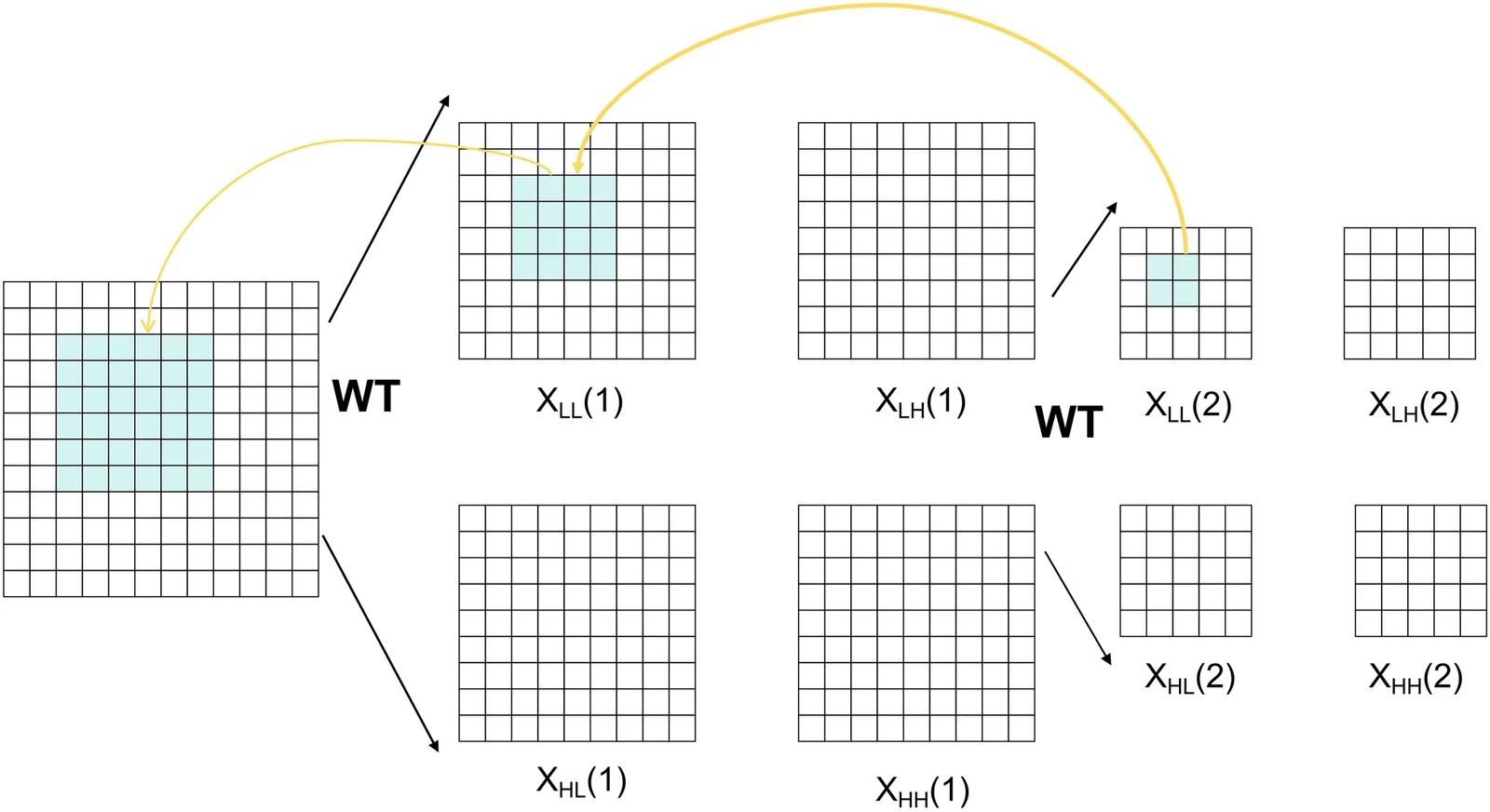
WT structure.

The WT-Conv module, as shown in Figure 4, processes input white blood cell images using the wavelet transform (WT). This decomposes the image into low-frequency and high-frequency components while performing filtering and downsampling operations to obtain image representations at different frequency bands. After acquiring these multi-frequency images, small-kernel depthwise convolution is applied to precisely extract more targeted features across each frequency dimension. Following the convolution operations, the inverse wavelet transform (IWT) reconstructs the frequency-specific images processed through convolution. This yields a final output that integrates multi-frequency features, enabling efficient and comprehensive extraction and synthesis of image characteristics from both multi-scale and multi-frequency perspectives. In this study, the WT-Conv module is instantiated at three feature extraction stages, with input and output channel configurations of 3→3, 64→64 and 128→128, respectively. Channel expansion between modules is performed by subsequent 1 × 1 convolutions, whilst spatial downsampling between stages is carried out by max-pooling. The three WT-Conv modules referred to here represent three invocation points within the network; this constitutes a different structural configuration from the number of wavelet decomposition layers within a single module, and the number of modules should not be directly interpreted as the number of wavelet decomposition layers.

**Figure 4:**
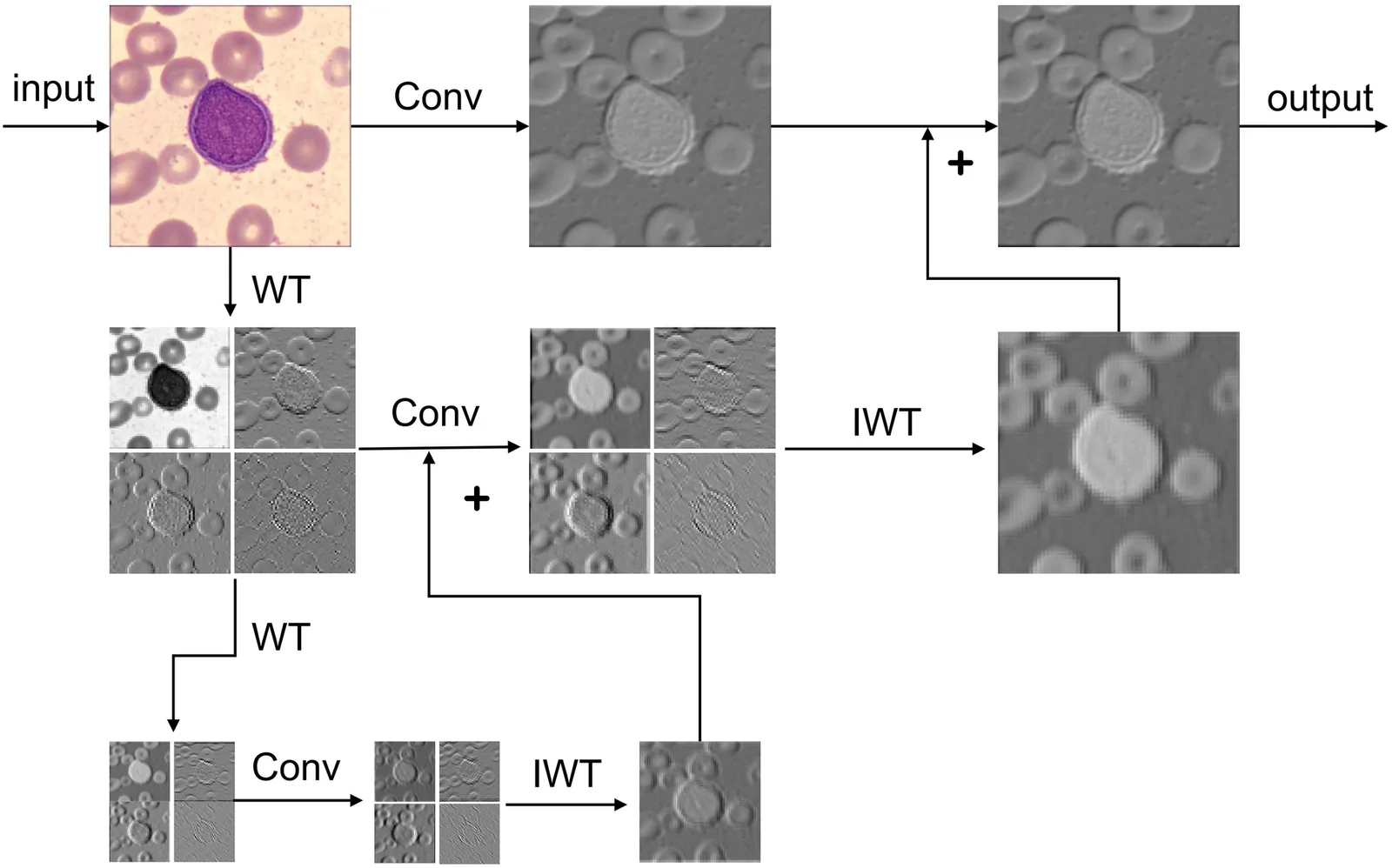
WT-Conv schematic diagram.

#### 3.2.2 ViT

Vision Transformer (ViT) is a variant of the Transformer architecture. Its core mechanism is the self-attention mechanism, which models relationships between patches and captures long-range dependencies in input data. ViT extends and applies the Transformer to computer vision. While traditional convolutional neural networks rely on local receptive fields for feature extraction, ViT treats images as a sequence of non-overlapping image patches, flattening these patches as input. ViT employs mechanisms similar to the Transformer, including Multi-Head Self-Attention (MHSA) and Positional Encoding (PosEnc), enabling the network to learn global feature representations without relying on convolutional operations. In ViT, the original image is first input. This 56×56 image undergoes convolution processing, transforming it into 4×4 patches of size 14×14. Each patch is then stretched into a 1×196 vector, combined with a 1×197 class_embedding vector, and merged with a 1×197 pos_embedding vector. This combined POS embedding vector is fed into an Encoder network, primarily based on the Transformer architecture, for training. Finally, for classification tasks, the output is the sum of the class embedding values across all patch vectors, which is then fed into a subsequent MLP for further computation. As shown in Figure 5.

**Figure 5:**
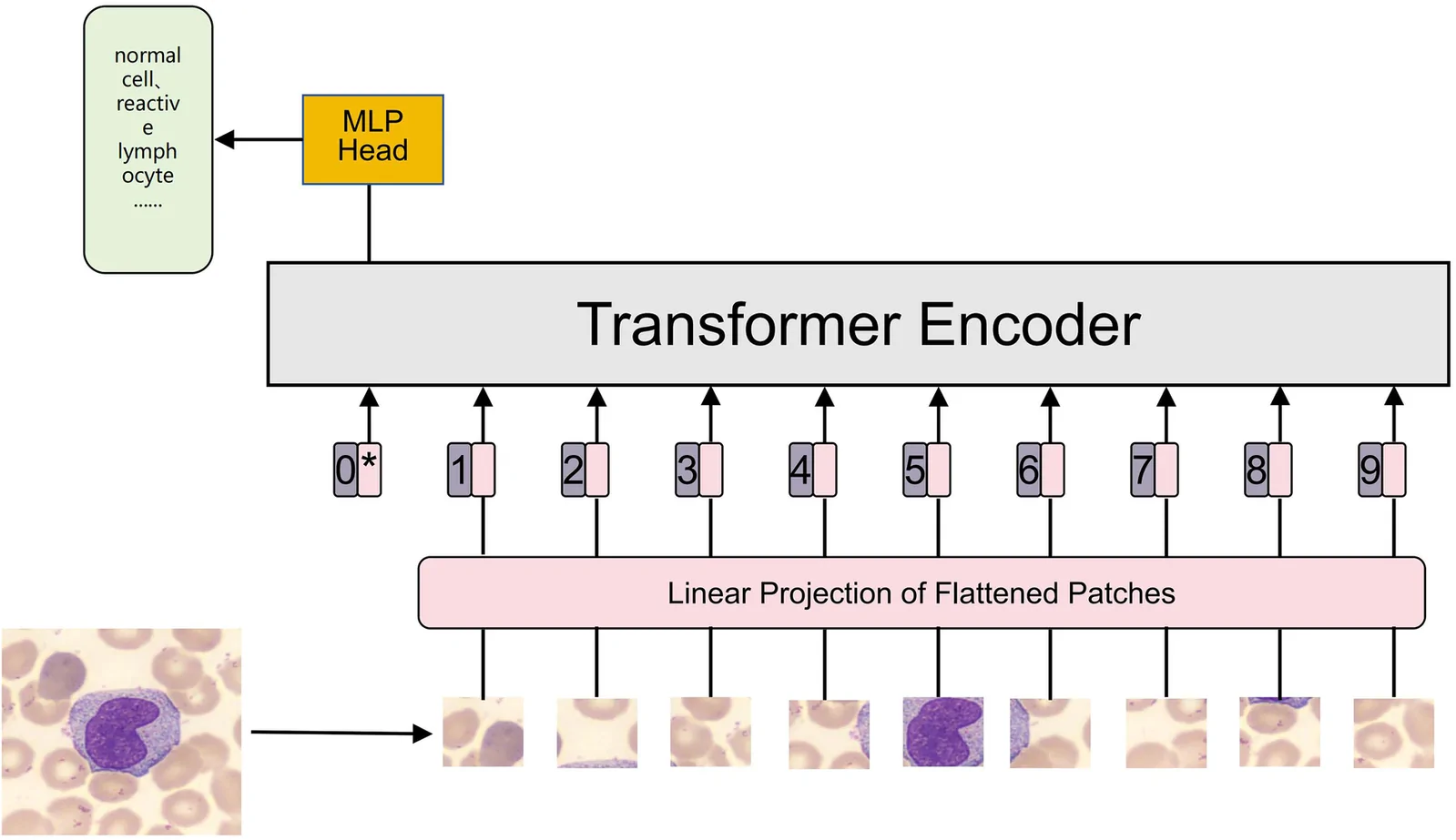
ViT schematic diagram.

This study introduces ViT, utilising its self-attention mechanism to model long-range dependencies and global feature correlations between different image regions, whilst integrating features extracted by the preceding Top-Hat, CDC and WT-Conv modules. Subsequently, ParNetAttention processes the fused features further. The impact of the aforementioned modules on classification metrics is assessed descriptively through the ablation experiments in Section 4.4. To clarify the actual block configuration, in this study, the 4 × 4 in the ViT module refers to the spatial dimensions of a single feature block, whilst 14 × 14 refers to the grid in which the feature blocks are arranged. The ViT receives fused features with 128 channels and spatial dimensions of 56 × 56, thereby forming a total of 196 non-overlapping feature blocks. Each feature block contains 4 × 4 × 128 = 2,048 elements, with the embedding dimension set to 1,024. The Transformer encoder has a depth of 1, 16 attention heads, and an MLP hidden dimension of 2,048, which is twice the embedding dimension. The dropout and emb_dropout parameters for the module are both set to 0.1. The num_classes parameter in the ViT constructor is set to 2,048; this parameter is configured separately from the final number of cell classification categories for the entire network; the output dimension of the final task is determined by the out_channels parameter of the terminal classification module. The above description clearly distinguishes between the feature block size, the number of feature blocks, the embedding dimension and the final classification output dimension.

#### 3.2.3 ParNetAttention attention

ParNetAttention is an efficient attention mechanism designed for sequence data, aiming to address the computational inefficiency of traditional attention mechanisms when processing long sequences. Traditional attention mechanisms compute attention weights by evaluating every position in the input sequence sequentially, resulting in high computational complexity for long sequences. This study introduces ParNetAttention following ViT to further process the fused features and provide feature representations for the subsequent classification module. The impact of this module on classification performance and computational overhead is described through a comparative analysis using the ablation settings and corresponding evaluation metrics established in this paper. The ParNet architecture hosting ParNetAttention has a depth of 12 and consists of parallel sub-structures. The width of each module is proportional to the output channel count of ParNet-M, while the height reflects the output resolution. Specifically, the ParNet module contains three parallel branches: a 1×1 convolution, a 3×3 convolution, and a Skip-Squeeze-Excitation (SSE) branch. In the workflow, input first enters these three parallel branches for feature extraction: the 1×1 convolution adjusts the number of channels, the 3×3 convolution captures local spatial features, and the SSE branch compresses channel-dimensional features into global descriptors via a squeeze operation, followed by an activation operation that assigns weights to each channel, achieving channel attention. After training, the 1×1 and 3×3 convolutions can be fused to accelerate inference speed. The SSE branch enhances feature attention capabilities by increasing the receptive field. In this study, ParNetAttention is instantiated with channel = 128 and placed after the ViT and before the final classification module. The final classification module comprises, in sequence, a 1 × 1 convolution, batch normalisation, a CELU activation function, adaptive global average pooling and a flattening operation. The 1 × 1 convolution maps the 128 input channels to K output channels, where K is the number of classes in the target task; adaptive average pooling reduces the spatial dimensions of each output channel to 1 × 1, and after flattening, this forms a K-dimensional classification output for each sample. The AWBC-LZWH and PBC_dataset tasks correspond to K = 6 and K = 8, respectively. In the provided model main programme and evaluation scripts, no additional convolution branch fusion or deployment conversion steps are explicitly called. Therefore, one cannot infer that the inference measurements in this paper have utilised a fused implementation solely on the basis that the ParNet architecture possesses the potential for branch fusion. Finally, features processed by each branch are fused to provide more discriminative features for subsequent operations like Avg pool + FC (average pooling followed by a fully connected layer), aiding classification tasks as shown in Figure 6.

**Figure 6:**
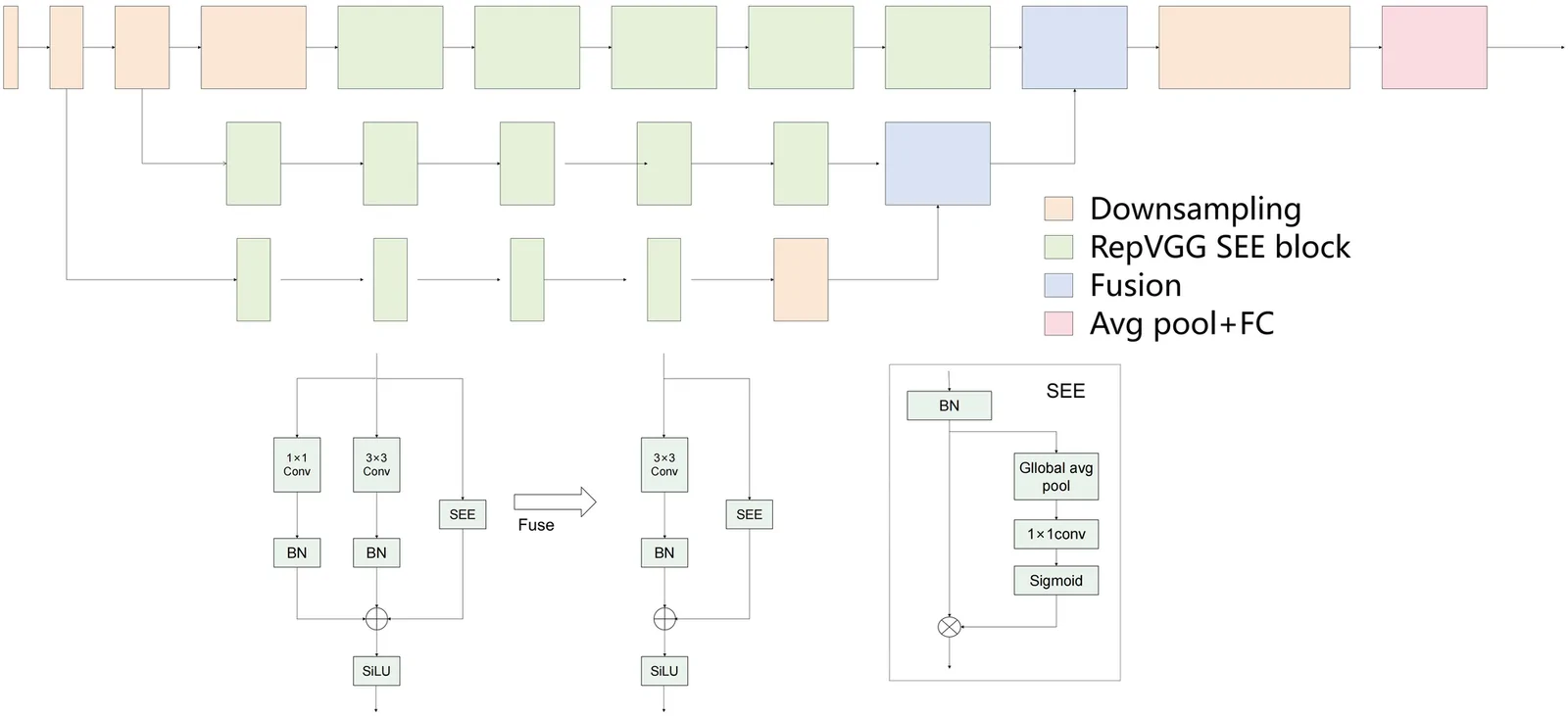
ParNetAttention schematic diagram.

## 4 Results

### 4.1 Experimental setup and environment

To validate the practical performance of the PV-WTConv model in abnormal white blood cell classification tasks, this paper selected the AWBC-LZWH dataset provided by the Laboratory Department of Liuzhou Workers’ Hospital as the experimental benchmark dataset. This dataset is used to evaluate the model’s classification performance on existing white blood cell images; it is not yet possible to draw conclusions about its performance in real-world clinical settings based on this data. During the model training phase, to ensure a stable and thorough training process, the total number of training epochs was set to 100, allowing the model sufficient iterations to learn the feature patterns within the data. Concurrently, refined settings were applied in both optimization strategy and data processing: A fixed learning rate of $1\times 10^{-4}$ was adopted. This value prevents initial over-high learning rates that cause training oscillations while avoiding excessively low rates later that slow convergence. The batch size was set to 16, balancing hardware computational load and training efficiency while ensuring stable gradient updates per epoch. Furthermore, to eliminate the interference of varying input image dimensions on model training, all raw images undergo uniform preprocessing. They are resized to the standard dimensions of 224×224 pixels, ensuring consistent data formats are input to the model. This lays the foundation for the model to efficiently extract features and accurately classify abnormal white blood cells. The development environment used for the experiments is shown in Table 2.

**Table 2:** Development environment.

Platform	Configuration
Integrated development environment	PyCharm
Scripting language	Python 3.9
Operating system	Windows 11
CPU	Intel Core i5-12400F
GPU	NVIDIA GeForce RTX 3060
Memory	32 GB
CUDA	11.6

In this study, patient-level stratified partitioning was performed at a ratio of 8:1:1 for the training, validation and test sets. The training set was used to optimise model parameters; the validation set was used to monitor performance during training and select the optimal weights; and the test set was used for the final performance evaluation once training and weight selection were complete. The metrics reported in this paper, such as ACC, Recall and F1-score, were all calculated using the model weights selected during the validation phase on the reserved image test set. For the AWBC-LZWH dataset, all models utilised the same fixed, patient-independent data partitioning. The 480 patients were divided in an 8:1:1 ratio into 384 training patients, 48 validation patients and 48 test patients; all cell images for a given patient were present in only one of these data subsets, and there was no overlap in patient identities across the three subsets. The training set was used for model parameter optimisation, the validation set for performance monitoring during training and the selection of optimal weights, whilst the test set was used solely for final performance evaluation once training and weight selection had been completed. Consequently, the ACC, Recall and F1-score reported in this paper were all calculated based on the cell images contained in the patient-independent test set. All networks under comparison utilised exactly the same patient partition to ensure consistency and fairness in the comparison between models. In this study, both the PV-WTConv and all comparison networks were trained independently from scratch on their respective datasets. They were not initialised using pre-trained weights from ImageNet or other datasets, nor were weights trained on one dataset used to initialise training on another. During training, the learnable parameters of both the feature extraction module and the classification head were included in the training process; the approach of freezing pre-trained layers followed by fine-tuning was not adopted. For the comparison networks, the basic structure of their original classification heads was retained, with only the number of classes in the final output layer adjusted according to the target task: 6 classes for the AWBC-LZWH task and 8 classes for the PBC_dataset task. The training set was used for parameter optimisation, whilst the validation set was used to monitor training performance and select model weights; the selected weights were then used for final evaluation on an independently reserved test set. To ensure the fairness of the comparative experiments, PV-WTConv, along with comparison models such as RegNet, GoogLeNet, ShuffleNet_v2_x1_0, DenseNet121, ResNet34, VGG16, EfficientNet, ResNet50 and VGG19 all employed the same data split, number of training epochs, learning rate, batch size, input dimensions, pre-processing and data augmentation strategies, and evaluation metrics were calculated on the same test set. All final classification metrics were calculated on the held-out test set; apart from differences in network architecture, all other training, validation and testing conditions remained consistent. Regarding data processing, all images were uniformly resized to 224 × 224 pixels before being fed into the network, converted to tensors and then normalised. During the training phase, only the training set underwent online data augmentation operations such as random flipping, random rotation and colour perturbation to improve generalisation ability; the validation and test sets underwent only deterministic pre-processing such as scaling, tensor transformation and normalisation, with no random augmentation introduced, to ensure stable and reproducible evaluation results, as shown in Table 3. To clarify how computational cost was measured, this study outlines the methods used for complexity analysis and performance statistics during the testing phase. The complexity analysis utilises THOP’s profile interface, with input consisting of a random tensor of shape 1 × 3 × 224 × 224, i.e., processing one three-channel image at a time. The statistical script divides the operation counts returned by the interface by $10^{9}$ for reporting purposes. This count follows THOP’s default operation counting rules, with convolution and linear layers using the “multiplication plus addition” counting method; the script does not perform any further standardised conversion to count multiplication and addition separately. The existing computational complexity profiling script does not explicitly provide supplementary counting rules for custom operators. In particular, the Top-Hat operation performed by OpenCV does not fall within the scope of PyTorch module operations counted by THOP; the coverage of statistical calculations for functional differentiation in CDC, as well as internal operations in modules such as the wavelet transform, also requires verification in conjunction with the corresponding implementations. Therefore, the tool’s return value should be interpreted as a complexity estimate for the operations it can recognise and must not be regarded directly as a complete estimate of computational cost covering all custom operations. The number of parameters is calculated by summing the number of elements in the model’s parameter tensors. The total number of parameters and the number of trainable parameters—as counted based on the requires_grad flag—are recorded separately; the parameter unit “M” denotes $10^{6}$ parameters. The count of trainable parameters reflects the parameter annotations of the current model instance and does not imply that gradients for every parameter have been verified as having been actually obtained during training. For FP32 parameters, the number of parameters multiplied by 4 and then divided by $1024^{2}$ yields the theoretical parameter storage requirement, expressed in MiB; this result must not be treated as the number of parameters expressed in M. Model size is calculated by reading the number of bytes in the saved model checkpoint used for testing and dividing it by $1024^{2}$, with the unit being MiB, where 1 MiB = 1,048,576 bytes. The training script provided uses the state_dict method to save model parameters and associated buffers; it does not save optimiser state. The actual file size and the theoretical storage capacity estimated based on the number of parameters are defined separately to avoid conflating the number of parameters, parameter storage capacity and file size as a single metric. During the testing phase, the input size is 224 × 224, the batch size is 16, and the number of data loading processes is 4; these batch settings are explained separately from the single-image inputs used in the complexity analysis. Before testing, model.eval() is called; the test loop is executed under torch.no_grad() conditions, with the cuda:0 device being prioritised when CUDA is available. Timing is measured using time.perf_counter(), starting before entering the test loop and ending after all test batches have been processed; when using CUDA, device synchronisation is performed at the start and end of the timing interval. This timing interval encompasses the loading of test data and associated waiting times, pre-processing, data transfer, network forward propagation, conversion of prediction results, calculation of classification metrics, and related processing within the loop. Model loading occurs prior to the start of timing, whilst the final confusion matrix is plotted and the results file is written after the timing has ended. The existing script does not include a separate warm-up phase, nor does it repeat the timing of the complete test loop multiple times. The average test time per image is calculated by dividing the total time of the complete test cycle by the number of images actually processed, then converting the result to milliseconds and multiplying by 1,000. Consequently, this metric represents the average time per image in the batch testing process, including the processing overhead of the test process itself. CPU memory usage is measured by reading the Resident Set Size (RSS) of the current main process via psutil. Samples are taken before and after the test cycle, and the difference between the post-test and pre-test values is recorded. These records represent pre-test memory, post-test memory and the change in memory, respectively; they do not represent peak CPU memory obtained from continuous sampling, nor do they include the total memory of all data-loading sub-processes. Where CUDA is available, the script also resets the peak GPU memory statistics before the test and records the peak allocated and peak reserved GPU memory of the PyTorch allocator after the test. CPU memory and GPU memory are reported separately, with the relevant byte counts divided by $1024^{2}$ and expressed in MiB. The learning rate adjustment function is called once after each optimiser parameter update within a mini-batch; when called, it is passed the current training epoch, the batch index within the epoch, and the total number of batches per epoch. Consequently, the scheduling calls in the code occur once per mini-batch; the specific pattern of learning rate changes also depends on the actual definition of the adjust_learning_rate function.

**Table 3:** Training parameters and hyperparameter settings.

Parameter	Value
Optimizer	AdamW
Loss function	Cross-entropy loss
Scheduler	adjust_learning_rate
Epochs	100
Learning rate	$10^{-4}$
Batch size	16
Weight decay	$10^{-5}$
Input size	224 × 224
Random seeds	2023, 2024, 2025, 2026, 2027
Number of workers	4

### 4.2 Evaluation criteria

This study employs a single-label, multi-class classification setting, in which the AWBC-LZWH and PBC_dataset contain 6 and 8 classes respectively. For each input image, the class with the highest predicted probability in the model’s output is taken as the final predicted label. This paper employs ACC, Recall, and F1-score as evaluation metrics to conduct comprehensive experimental comparisons. Specifically, these evaluation metrics are defined as follows: Accuracy: Accuracy is used to measure the proportion of images where the predicted class matches the true class, rather than the average of the “one-vs.-all” binary classification accuracies for each class. As a fundamental and commonly used evaluation metric in classification tasks, it is a core indicator for measuring model performance by calculating the degree of agreement between the model’s predictions and the true labels in the data, as shown in Equation 3.

$$\mathrm{ACC}=\frac{\mathrm{TP}+\mathrm{TN}}{\mathrm{TP}+\mathrm{FP}+\mathrm{FN}+\mathrm{TN}}$$ (3)

Recall is calculated starting from the true positive labels, representing the proportion of correctly predicted positive samples (True Positives, TP) out of all true positive samples (TP + False Negatives, FN), as defined in Equation 4.

$$\text{Recall }=\frac{\mathrm{TP}}{\mathrm{TP}+\mathrm{FN}}$$ (4)

The F1-score is a metric used to evaluate the performance of classification models, and is particularly suitable for situations where positive and negative samples are imbalanced. It combines precision and recall, as defined in Equation 5.

F1=2×Precision×RecallPrecision+Recall=2TP2TP+FP+FN (5)

### 4.3 Experimental results

Repeated experiments with different random seeds utilise the same training, validation and test sets; the data subsets are determined by a pre-defined directory, and the training script does not re-execute data partitioning when the seed changes. Variations in the random seed affect random operations within the model, such as the initialisation of random parameters, sample shuffling during data loading, random data augmentation during the training phase, and dropout. Therefore, different seeds correspond to variations in training randomness under a fixed data partition, rather than different data partitioning schemes. The means and standard deviations reported in this paper are used to describe variations in metrics across the aforementioned repeated experiments; they do not imply that statistical significance tests have been conducted on performance differences between models. This study employs a descriptive approach to model comparison, and the relevant conclusions are limited to the specific datasets, data partitions and training settings used; they do not constitute a claim that any model possesses a statistically significant advantage. To further enhance the reliability and completeness of the experimental results, this paper has added four sets of experiments using random seeds 2024, 2025, 2026 and 2027 to the original 2023 random seed experiments, and reports model performance in the form of the mean and standard deviation of the five experimental results. Furthermore, to broaden the comparison with traditional deep learning architectures, this paper has added three classic networks—ResNet18, ResNet101 and MobileNet—to the existing set of comparison models. Three additional evaluation metrics—inference time, CPU memory usage and model size—have also been introduced to provide a more comprehensive assessment of the differences between various models in terms of classification performance, stability and deployment efficiency. Table 4 summarises the complete comparison results on the AWBC-LZWH dataset, whilst Table 5 further presents the results of the additional generalisation experiments conducted on the PBC_dataset. This study evaluated PV-WTConv against 12 comparison models on the AWBC-LZWH dataset; the relevant results are shown in Table 4. Under the data split and training settings adopted, PV-WTConv achieved an average accuracy, F1-score and recall of 94.4%, 93.7% and 94.3% respectively, which were the highest among the corresponding mean metrics of the comparison models in the table. In particular, its average accuracy was 3.0 percentage points higher than the 91.4% achieved by ResNet50. These results describe differences in mean performance observed under the current experimental conditions. No formal statistical tests were conducted to assess the significance of these differences. Figure 7 shows the class recognition results and misclassification distributions for each model on the test set, which can be used to observe differences in performance across categories. Figure 8 illustrates the changes in accuracy and loss during the training and validation phases, describing the convergence process of each model under the current settings. These graphical results should be interpreted in conjunction with the evaluation metrics from the test set; they cannot be used in isolation to conclude that a particular model is superior across all categories or possesses stronger generalisation ability.

**Table 4:** Comparison of experimental results for different algorithms on the AWBC-LZWH dataset.

Name	FLOPS (G)	Params (M)	F1-score	Recall	ACC	Inference time (ms/image)	CPU memory usage (M)	Model size (M)
PV-WTConv	4.29	12.92	0.937±0.022	0.943±0.017	0.944±0.015	48.82	3,659.82	51.24
RegNet	0.43	5.10	0.847±0.032	0.861±0.031	0.880±0.027	30.32	3,586.42	20.43
DenseNet121	2.90	69.60	0.874±0.026	0.889±0.024	0.895±0.023	48.37	3,652.52	278.84
EfficientNet	0.41	4.02	0.882±0.022	0.902±0.020	0.890±0.025	30.02	3,585.31	16.11
GoogLeNet	15.10	56.10	0.850±0.033	0.865±0.034	0.878±0.028	44.59	3,638.69	224.75
ResNet34	3.68	21.29	0.872±0.026	0.887±0.025	0.896±0.022	34.85	3,603.01	85.29
ResNet50	4.13	23.52	0.899±0.021	0.914±0.019	0.914±0.018	35.47	3,605.30	94.23
ShuffleNet_v2_x1_0	1.51	125.90	0.887±0.027	0.903±0.026	0.910±0.024	64.13	3,670.22	504.39
VGG16	15.47	134.29	0.806±0.034	0.830±0.036	0.840±0.033	66.48	3,678.82	538.01
VGG19	19.63	196.28	0.789±0.037	0.806±0.034	0.834±0.030	83.83	3,682.35	786.36
ResNet18	1.82	10.67	0.871±0.028	0.884±0.025	0.894±0.027	32.62	3,591.81	42.73
ResNet101	7.86	40.64	0.903±0.018	0.926±0.014	0.922±0.017	32.59	3,588.47	162.81
MobileNet	0.33	2.17	0.877±0.019	0.881±0.023	0.931±0.012	30.30	3,589.98	8.70

**Table 5:** Comparison of experimental results for different algorithms on the PBC_dataset.

Name	FLOPS (G)	Params (M)	F1-score	Recall	ACC	Inference time (ms/image)	CPU memory usage (M)	Model size (M)
PV-WTConv	4.29	12.92	0.962±0.022	0.967±0.019	0.971±0.017	13.55	3,659.82	51.24
RegNet	0.43	5.10	0.812±0.035	0.830±0.033	0.875±0.030	12.02	3,590.75	20.43
DenseNet121	2.90	69.60	0.844±0.029	0.856±0.027	0.890±0.028	12.63	3,595.92	278.84
EfficientNet	0.41	4.02	0.757±0.037	0.781±0.036	0.821±0.031	12.01	3,590.67	16.11
GoogLeNet	15.10	56.10	0.807±0.033	0.824±0.035	0.862±0.028	14.03	3,594.84	224.75
ResNet34	3.68	21.29	0.828±0.031	0.841±0.032	0.883±0.026	12.49	3,592.05	85.29
ResNet50	4.13	23.52	0.921±0.023	0.929±0.020	0.941±0.016	12.55	3,592.23	94.23
ShuffleNet_v2_x1_0	1.51	125.90	0.890±0.026	0.898±0.023	0.925±0.019	12.75	3,680.43	504.39
VGG16	15.47	134.29	0.633±0.039	0.667±0.034	0.700±0.031	14.47	3,681.11	538.01
VGG19	19.63	196.28	0.553±0.036	0.584±0.037	0.640±0.034	15.28	3,686.07	786.36
ResNet18	1.82	10.67	0.919±0.024	0.906±0.026	0.931±0.023	11.67	3,593.20	42.73
ResNet101	7.86	40.64	0.943±0.018	0.933±0.017	0.947±0.015	12.90	3,591.01	162.81
MobileNet	0.33	2.17	0.947±0.012	0.937±0.011	0.951±0.013	12.21	3,591.20	8.70

**Figure 7:**
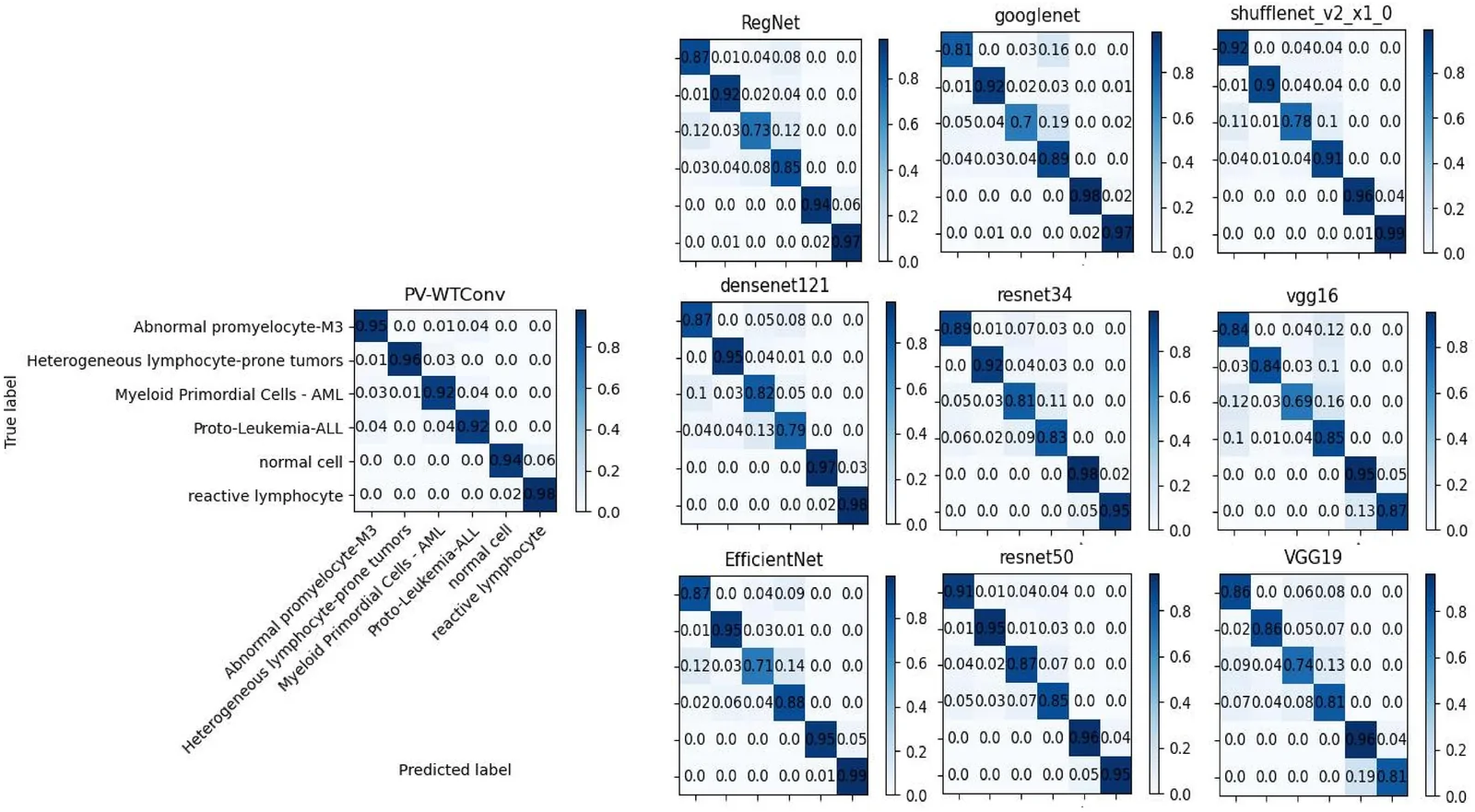
Comparison chart of confusion matrices for experiments.

**Figure 8:**
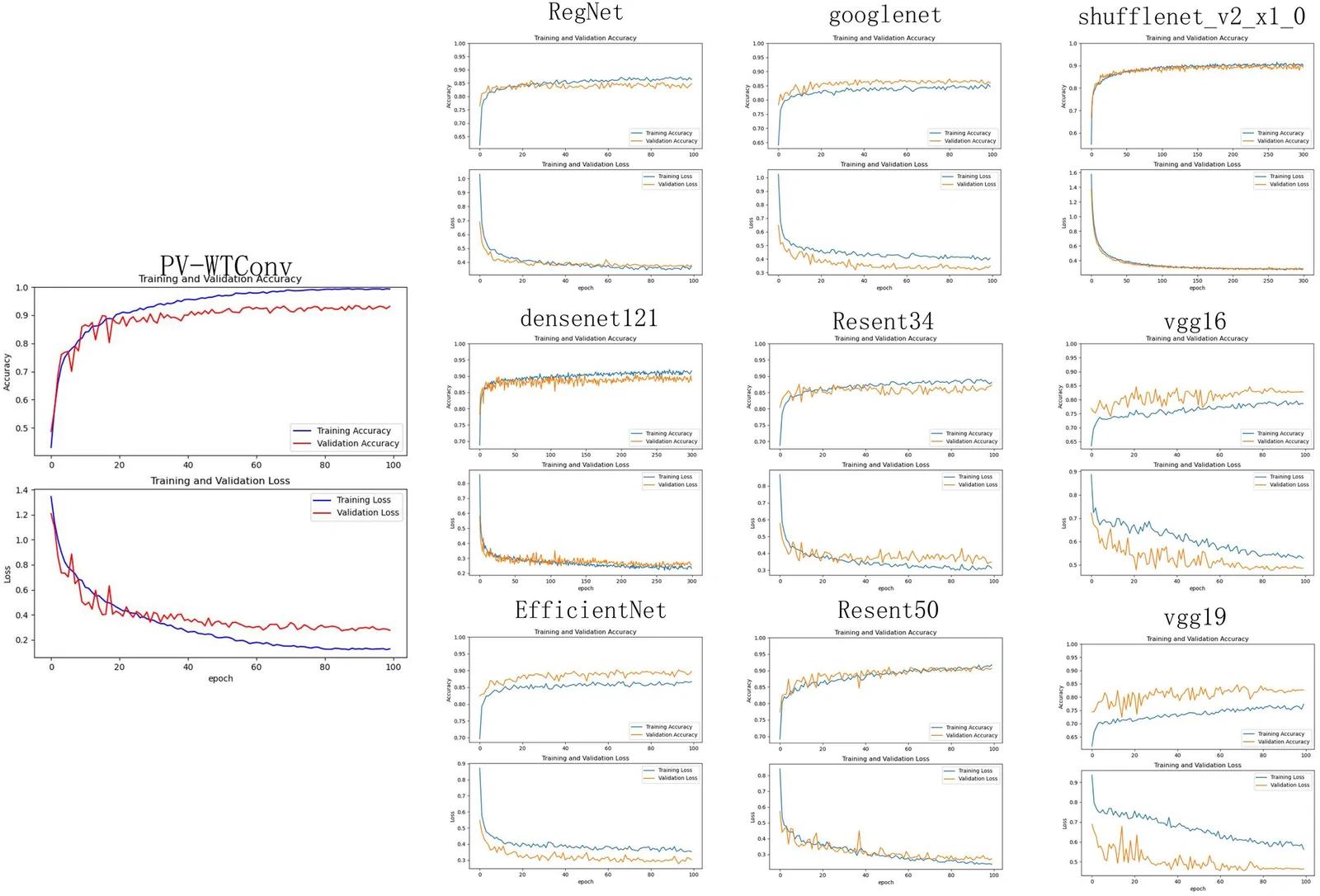
Comparison of experimental accuracy and loss curve changes.

To further evaluate the adaptability of the PV-WTConv network architecture to different blood cell classification tasks, this study conducted an independent eight-class classification experiment on the publicly available PBC_dataset. Unlike the six-class classification task in AWBC-LZWH, the PBC_dataset comprises eight classes: neutrophils, eosinophils, basophils, lymphocytes, monocytes, immature granulocytes, erythroblasts and platelets. Consequently, the experiments on the PBC_dataset serve to evaluate the classification performance of the same network architecture under a different classification scheme, and should not be interpreted as external validation of the original six-class classifier from AWBC-LZWH. All comparison networks had their output layers reconfigured in accordance with the eight-class classification task of the PBC_dataset, and were trained and evaluated independently under the same training, validation and testing conditions; the results are shown in Table 5. Table 5 summarises the classification results and computational overhead of each model on the PBC_dataset. Under the current experimental setup, PV-WTConv achieved an average accuracy, F1-score and recall of 97.1%, 96.2% and 96.7%, respectively, which are 3.0, 4.1 and 3.8 percentage points higher than the corresponding averages of ResNet50. These differences reflect the average performance of the evaluated models in this eight-class classification task; their statistical significance has not yet been tested. In terms of computational overhead, PV-WTConv reports a computational cost of 4.29 G, which is close to ResNet50’s 4.13 G and lower than the corresponding figures for GoogLeNet, VGG16 and VGG19 shown in the table; it has 12.92 M parameters. Computational effort, number of parameters, inference time and memory usage reflect different aspects of computational requirements. These metrics describe the trade-off between classification performance and computational cost under the evaluated settings. These results may serve as a reference for subsequent deployment studies, although the feasibility of deployment on specific devices still requires further evaluation.

Figure 9 shows the class-wise classification results and misclassification distributions for each model on the PBC_dataset. In the confusion matrix shown, the correct classification rates for each class in the PV-WTConv model range between 0.93 and 1.00, though some confusion still exists between certain classes. The misclassification distributions vary across different models; these results can be used to characterise the class-wise classification performance on the current test data. The confusion matrix alone cannot establish the statistical significance of differences between models, nor can it be used to determine whether a particular model meets or fails to meet clinical diagnostic requirements.

**Figure 9:**
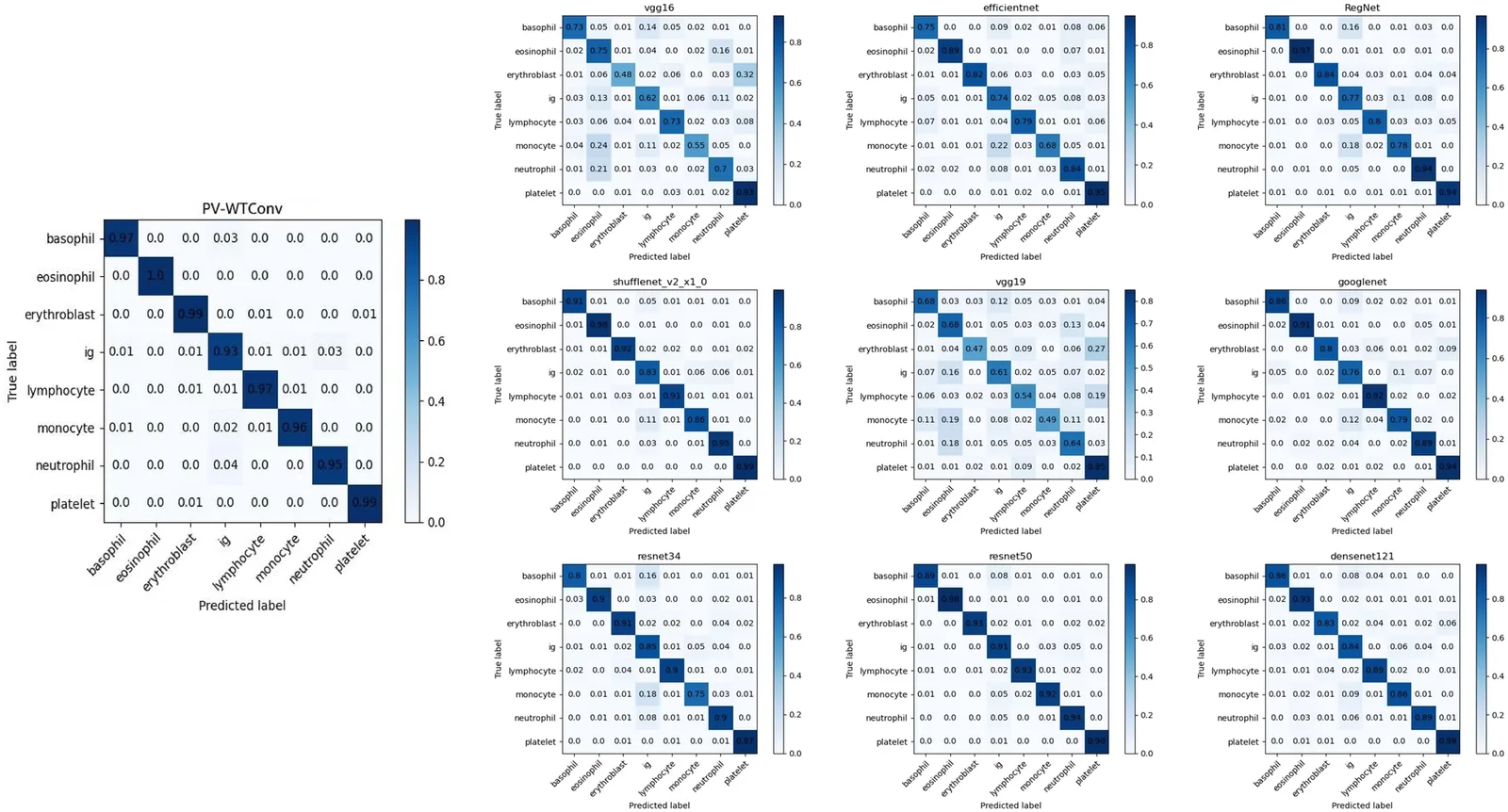
Comparison chart of confusion matrices for public dataset experiments.

Figure 10 illustrates the training and validation accuracy, as well as the changes in loss, for each model on the PBC_dataset. The PV-WTConv curve shown exhibits a trend of rising accuracy and decreasing loss in the early stages of training, before gradually levelling off as training progresses. Other comparison models exhibit different patterns of accuracy changes, loss levels and fluctuations. These graphical results serve to describe the training and validation behaviour observed under the current experimental setup and do not constitute proof of statistical significance regarding differences in convergence speed, training stability or generalisation ability. The corresponding test-set classification results are reported in Table 5.

**Figure 10:**
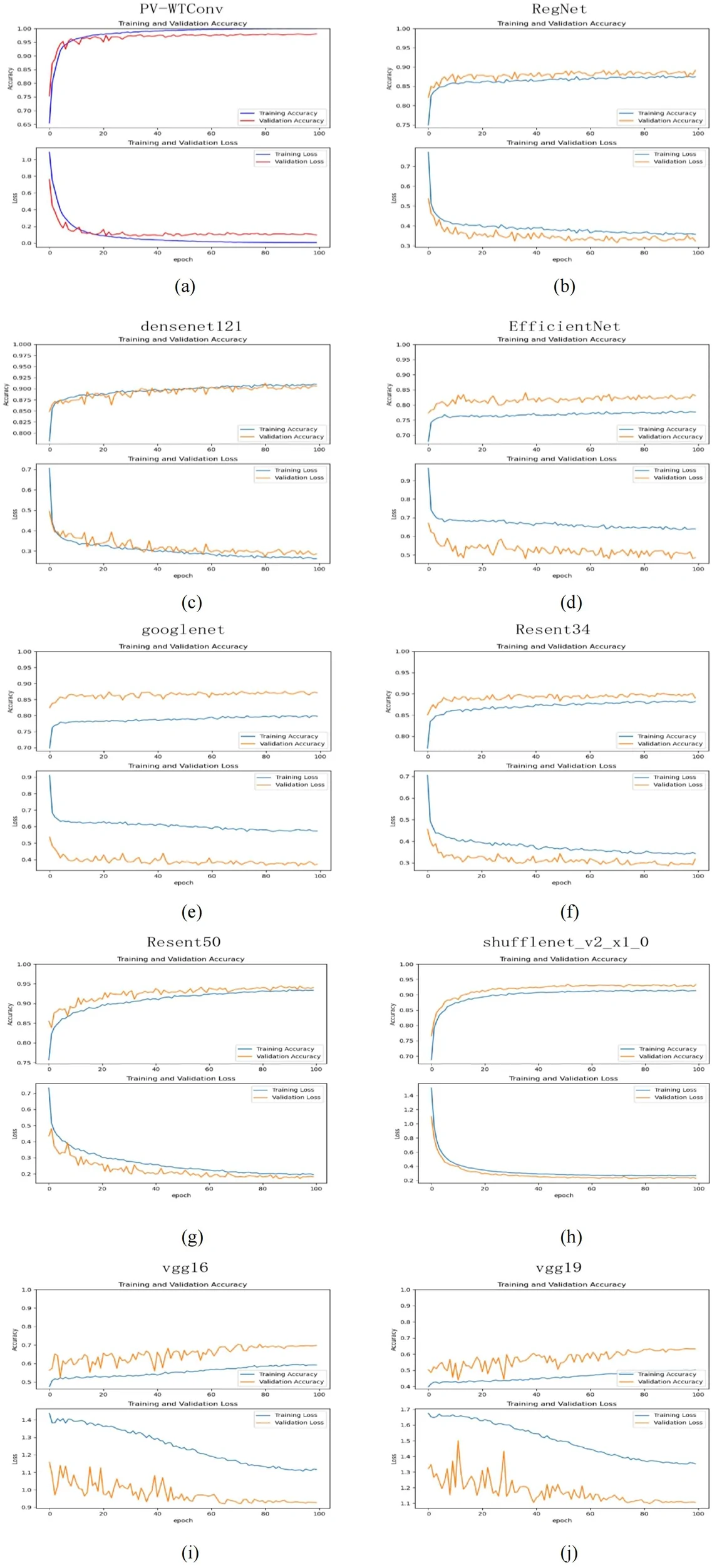
Comparison of changes in accuracy and loss curves in public dataset experiments. Panels **(a)**–**(j)** show the experimental accuracy and loss curves for the PV-WTConv, RegNet, DenseNet121, EfficientNet, GoogLeNet, ResNet34, ResNet50, ShuffleNet_v2_x1_0, VGG16 and VGG19 networks, respectively.

Building upon the aforementioned experiments on the independent eight-class classification task, and in order to further investigate the feature transfer capabilities of different networks across datasets and tasks, this study additionally conducted cross-dataset experiments involving transfer from the PBC_dataset to the AWBC-LZWH task. Specifically, the weights trained by each model on the PBC_dataset eight-class classification task were first loaded as parameter initialisations. Whilst retaining the learned feature representations, the final classification output layer was adjusted to correspond to the six classes of the AWBC-LZWH task, and the models were adapted to the target task using the AWBC-LZWH training data, with evaluation subsequently carried out on its reserved test set. All models were subjected to consistent data processing, training and evaluation conditions; experiments were repeated using different random seeds, and results are presented as mean ± standard deviation. As shown in Table 6, in this cross-dataset transfer experiment, PV-WTConv achieved an F1-score of 0.934±0.018, a recall of 0.933±0.016 and an ACC of 0.931±0.015. Compared with ResNet50, its F1-score, Recall and ACC improved by 4.9, 3.2 and 2.6 percentage points, respectively. Among the models compared in Table 6, PV-WTConv achieved the highest mean F1-score and recall; its mean accuracy equalled the reported mean accuracy of MobileNet at 0.931. These results describe the performance of the various models on the target task’s test set following adaptation to the AWBC-LZWH training data, providing descriptive evidence of cross-task transfer under the current settings.

**Table 6:** Comparison of cross-dataset transfer results for different algorithms from the eight-class PBC_dataset task to the six-class AWBC-LZWH task.

Name	FLOPS (G)	Params (M)	F1-score	Recall	ACC	Inference time (ms/image)	CPU memory usage (M)	Model size (M)
PV-WTConv	4.29	12.92	0.934±0.018	0.933±0.016	0.931±0.015	13.84	3,649.35	51.24
RegNet	0.43	5.10	0.835±0.031	0.854±0.029	0.869±0.026	7.63	3,584.76	20.43
DenseNet121	2.90	69.60	0.848±0.027	0.869±0.023	0.881±0.021	9.22	3,575.72	278.84
EfficientNet	0.41	4.02	0.871±0.020	0.889±0.017	0.876±0.015	9.11	3,580.19	16.11
GoogLeNet	15.10	56.10	0.835±0.035	0.857±0.032	0.861±0.029	8.43	3,587.35	224.75
ResNet34	3.68	21.29	0.863±0.025	0.876±0.023	0.883±0.020	9.89	3,596.69	85.29
ResNet50	4.13	23.52	0.885±0.022	0.901±0.017	0.905±0.016	10.15	3,593.19	94.23
ShuffleNet_v2_x1_0	1.51	125.90	0.865±0.028	0.891±0.023	0.898±0.021	10.37	3,680.43	504.39
VGG16	15.47	134.29	0.802±0.033	0.821±0.032	0.828±0.030	15.35	3,690.64	538.01
VGG19	19.63	196.28	0.784±0.035	0.799±0.032	0.817±0.028	15.72	3,686.07	786.36
ResNet18	1.82	10.67	0.857±0.032	0.875±0.028	0.869±0.025	11.98	3,586.58	42.73
ResNet101	7.86	40.64	0.885±0.019	0.915±0.016	0.911±0.015	12.79	3,591.01	162.81
MobileNet	0.33	2.17	0.877±0.019	0.881±0.023	0.931±0.012	8.47	3,591.20	8.70

It should be emphasised that this experiment constitutes an evaluation of transferability across datasets and classification schemes, rather than an external validation of the original AWBC-LZWH six-classification model. As the target categories in the PBC_dataset do not correspond to those in AWBC-LZWH, true external validation of the original six-classification model still requires the use of a patient or sample cohort containing the same six target categories and independent of the model’s training data, with the six-classification model trained on AWBC-LZWH being directly applied to this independent cohort for evaluation.

### 4.4 Ablation experiment

In this study, using the AWBC-LZWH dataset, we conducted a comprehensive evaluation of the performance of each model within the proposed network architecture. To analyse the impact of different model components on overall performance, this study conducted ablation experiments using a serial convolutional network as the baseline. During the experiments, specific network modules were progressively removed or replaced, and the performance before and after these structural changes was compared to systematically evaluate the contribution of each component to the model’s overall performance. The ablation experiment results of different module combinations on the AWBC-LZWH dataset are shown in Table 7.

**Table 7:** Ablation experiment results of different module combinations on the AWBC-LZWH dataset.

Name	FLOPS (G)	Params (M)	F1-score	Recall	ACC	Inference time (ms/image)	CPU memory usage (M)	Model size (M)
Conv	0.11	0.01	0.485±0.023	0.457±0.021	0.569±0.016	29.81	3739.20	0.04
Conv+Top-Hat	0.11	0.01	0.509±0.027	0.496±0.024	0.597±0.019	38.66	3759.16	0.04
Conv+CDC	1.13	0.08	0.521±0.033	0.517±0.029	0.665±0.028	33.24	3736.63	0.33
Conv+WT-Conv	0.18	0.03	0.538±0.026	0.525±0.025	0.679±0.022	30.88	3739.14	0.17
Conv+ViT	2.59	12.61	0.613±0.018	0.676±0.015	0.763±0.013	32.36	3737.46	48.87
Conv+ParNetAttention	0.63	0.19	0.594±0.020	0.620±0.017	0.727±0.014	30.23	3736.93	0.74
Conv+Top-Hat+CDC	1.13	0.08	0.598±0.024	0.621±0.023	0.735±0.020	43.05	3760.70	0.33
Conv+Top-Hat+WT-Conv	0.18	0.03	0.585±0.037	0.616±0.035	0.724±0.031	39.13	3754.33	0.17
Conv+Top-Hat+ViT	2.59	12.61	0.782±0.023	0.776±0.020	0.851±0.019	41.14	3659.82	48.87
Conv+Top-Hat+ParNetAttention	0.63	0.19	0.605±0.029	0.632±0.025	0.745±0.022	39.39	3759.37	0.74
Conv+CDC+WT-Conv	1.19	0.11	0.741±0.032	0.743±0.029	0.809±0.026	33.25	3740.86	0.46
Conv+CDC+ViT	3.61	12.68	0.802±0.028	0.799±0.024	0.868±0.021	31.83	3738.03	49.16
Conv+CDC+ParNetAttention	1.64	0.27	0.761±0.025	0.756±0.018	0.836±0.016	32.31	3737.04	1.03
Conv+WT-Conv+ViT	2.66	12.63	0.809±0.017	0.803±0.016	0.871±0.014	31.48	3736.12	49.00
Conv+WT-Conv+ParNetAttention	0.70	0.22	0.794±0.035	0.786±0.031	0.856±0.030	30.83	3739.70	0.87
Conv+ViT+ParNetAttention	3.11	12.79	0.887±0.023	0.871±0.019	0.914±0.017	31.59	3737.16	49.57
Conv+Top-Hat+CDC+ViT	3.61	12.68	0.874±0.027	0.858±0.023	0.909±0.021	42.94	3759.14	49.16
Conv+Top-Hat+CDC+ParNetAttention	1.64	0.27	0.786±0.021	0.764±0.017	0.841±0.015	39.36	3759.63	1.03
Conv+Top-Hat+CDC+WT-Conv	1.19	0.11	0.812±0.030	0.808±0.026	0.875±0.023	40.22	3755.34	0.46
Conv+Top-Hat+WT-Conv+ViT	2.66	12.63	0.856±0.033	0.838±0.028	0.891±0.024	40.14	3753.70	49.01
Conv+Top-Hat+WT-Conv+ParNetAttention	0.70	0.22	0.844±0.031	0.826±0.029	0.881±0.026	39.57	3756.35	0.87
Conv+Top-Hat+ViT+ParNetAttention	3.11	12.79	0.892±0.016	0.874±0.015	0.916±0.013	39.23	3761.43	49.58
Conv+CDC+WT-Conv+ViT	3.67	12.71	0.870±0.022	0.855±0.020	0.908±0.019	34.40	3735.85	49.29
Conv+CDC+WT-Conv+ParNetAttention	1.71	0.29	0.863±0.030	0.847±0.027	0.895±0.023	34.08	3735.37	1.16
Conv+CDC+ViT+ParNetAttention	4.12	12.86	0.887±0.018	0.872±0.015	0.915±0.013	37.77	3738.94	49.86
Conv+WT-Conv+ViT+ParNetAttention	3.18	12.81	0.889±0.017	0.879±0.016	0.931±0.014	32.73	3739.19	49.71
Conv+Top-Hat+CDC+WT-Conv+ViT	3.67	12.71	0.874±0.021	0.869±0.018	0.920±0.017	41.15	3751.93	49.29
Conv+Top-Hat+CDC+WT-Conv+ParNetAttention	1.71	0.29	0.871±0.025	0.867±0.022	0.919±0.019	40.69	3758.02	1.16
Conv+Top-Hat+CDC+ViT+ParNetAttention	4.12	12.86	0.891±0.016	0.876±0.014	0.928±0.013	40.01	3761.11	49.86
Conv+Top-Hat+WT-Conv+ViT+ParNetAttention	3.18	12.81	0.897±0.020	0.883±0.018	0.932±0.015	41.89	3755.20	49.71
Conv+CDC+WT-Conv+ViT+ParNetAttention	4.19	12.89	0.902±0.023	0.888±0.021	0.934±0.017	32.69	3738.74	49.99
Conv+Top-Hat+CDC+WT-Conv+ViT+ParNetAttention	4.29	12.92	0.937±0.022	0.943±0.017	0.944±0.015	48.82	3659.82	51.24

To further assess the contributions of the Top-Hat, WT-Conv, CDC, ViT and ParNetAttention modules to the performance of the PV-WTConv network, this paper conducts ablation experiments on the AWBC-LZWH dataset. All ablation configurations utilised the same data split, training parameters and pre-processing methods, and were repeated five times using 2023, 2024, 2025, 2026 and 2027 as random seeds, respectively. The ACC, F1-score and Recall values in Table 7 are presented as the mean ± standard deviation of the five runs, in order to illustrate both the average classification performance of each configuration and the inter-run variability caused by different random seeds. The experiments used a Conv network comprising only traditional convolutions as the baseline model. Building upon this, individual modules, multiple modules and complete combinations of modules were introduced in turn. By comparing metrics such as F1, Recall, ACC, FLOPs, number of parameters, inference time, CPU memory usage and model size, we systematically analysed the impact of each module on the performance of abnormal white blood cell classification. Table 7 presents the results of the ablation experiments for different module combinations on the AWBC-LZWH dataset. As shown in Table 7, the baseline Conv model achieved an ACC of 56.9%, an F1-score of 48.5% and a Recall of 45.7%. Under the current experimental setup, when Top-Hat, WT-Conv, CDC, ViT or ParNetAttention were added respectively, the average ACC, F1-score and recall for each configuration were higher than those of the Conv baseline. These results illustrate the differences in average classification performance between the evaluated configurations. Specifically, Top-Hat enhances bright regions and morphological details of the cells; WT-Conv aids in extracting multi-scale frequency-domain features; CDC further emphasises local texture and edge differences; ViT models global dependencies between different regions; and ParNetAttention enhances the expression of key features. Furthermore, when examining the results of removing individual modules from the complete model, removing Top-Hat, CDC, WT-Conv, ViT and ParNetAttention reduced the mean accuracy from 94.4% to 93.4%, 93.2%, 92.8%, 91.9% and 92.0%, respectively, corresponding to decreases of 1.0, 1.2, 1.6, 2.5 and 2.4 percentage points. Based on the aggregated results from five runs, the ACC, F1-score and Recall of the complete model were 0.944 ± 0.015, 0.937 ± 0.022 and 0.943 ± 0.017, respectively; the mean values for all three metrics were higher than those for configurations in which Top-Hat, WT-Conv, CDC, ViT or ParNetAttention were removed individually. These results support the contribution of the aforementioned modules to the average classification performance under the current experimental setup. For smaller differences—such as the 1.0 percentage point decrease in average ACC following the removal of Top-Hat—interpretation should be approached with caution, taking into account the corresponding standard deviation, and the difference in mean values should not be directly regarded as evidence of statistical significance.

## 5 Discussion

### 5.1 Analysis of results

The PV-WTConv model proposed in this paper constructs a feature representation for the classification of abnormal white blood cells by combining morphological, frequency-domain, local texture and global correlation information. On the AWBC-LZWH dataset, its average accuracy, F1-score and recall were 94.4%, 93.7% and 94.3% respectively, which were 3.0, 3.8 and 2.9 percentage points higher than the corresponding averages of ResNet50. In the independent eight-class classification experiment on the PBC_dataset, the corresponding mean values were 97.1%, 96.2% and 96.7%, which were 3.0, 4.1 and 3.8 percentage points higher than those of ResNet50, respectively. The above results describe the average performance of this architecture across the two classification tasks under the current experimental setup. The observed differences may be related to the feature modelling approach adopted; however, the comparison results alone are insufficient to establish this mechanism as an explanation, nor do they constitute evidence of universal superiority or statistically significant improvements in the model. The ablation experiments in Section 4.4 provide a descriptive assessment of the contributions of these modules. In terms of computational overhead, the reported computational cost of PV-WTConv is approximately 4.29 G, which is close to the 4.13 G of ResNet50 and lower than the corresponding figures for GoogLeNet, VGG16 and VGG19 in the table. The classification metrics and computational cost reported here describe the performance—cost trade-off under the current experimental settings.

### 5.2 ROC curve analysis

To further illustrate the classification performance of PV-WTConv and the various comparison models on the AWBC-LZWH dataset, this paper plots the corresponding ROC curves and reports the AUC values, as shown in Figure 11. The ROC curve, with the false positive rate (FPR) on the x-axis and the true positive rate (TPR) on the y-axis, intuitively reflects the model’s classification performance at different classification thresholds, whilst the AUC value quantifies the model’s overall classification capability; the closer the value is to 1, the better the classification performance. Figure 11 shows the macro-average and per-category ROC curves for each model on the AWBC-LZWH dataset. PV-WTConv achieved a macro-average AUC of 0.9976 in this evaluation. The macro-average curve is used to summarise category discrimination performance, whilst the per-category curves are used to observe the true positive rate and false positive rate for different categories as the classification threshold varies. The comparison of ROC curves and AUCs in this paper is presented for descriptive purposes only; it does not constitute a determination of statistically significant differences between models. Furthermore, the appearance of the curves and the AUC results alone cannot prove the stability of the models across different data distributions or their clinical diagnostic validity.

**Figure 11:**
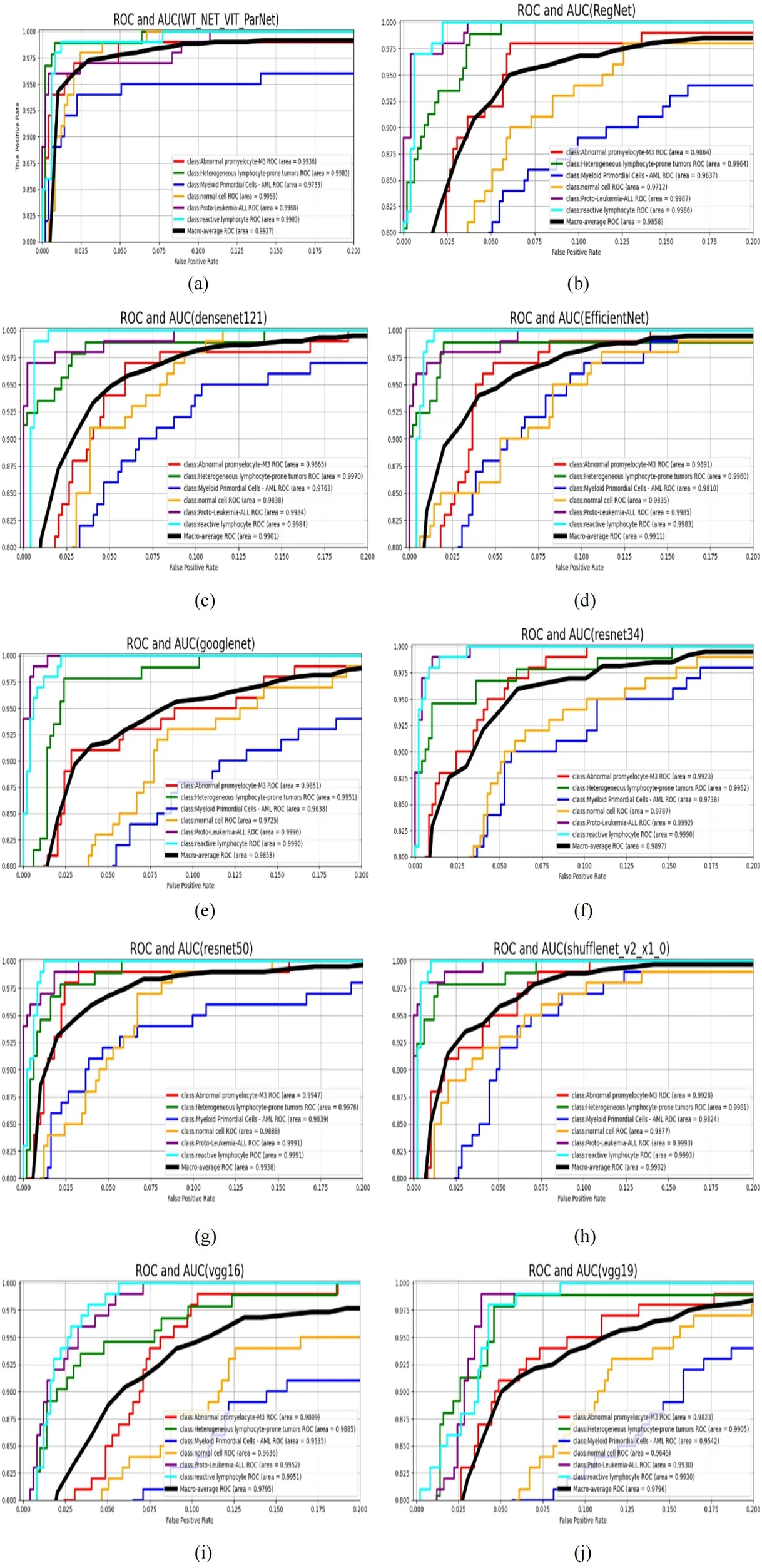
Comparison of ROC curves in comparative experiments **(a)**–**(j)** are the ROC curves of the PV-WTConv, RegNet, DenseNet121, EfficientNet, GoogLeNet, ResNet34, ResNet50, ShuffleNet_v2_x1_0, VGG16 and VGG19 networks, respectively.

Figure 12 shows the ROC curves and corresponding AUC results for each model on the PBC_dataset; PV-WTConv reported an AUC of 0.9986 in this evaluation. The ROC curves for each class are used to illustrate the classification performance of the corresponding class at different thresholds, supplemented by accuracy, recall and F1-scores under a fixed classification rule. Differences in the positions of the curves and AUC values between models are presented here as descriptive observations; they should not be interpreted as advantages supported by statistical testing, nor should they be used to infer that the models exhibit identical performance across all thresholds or under all application conditions (32).

**Figure 12:**
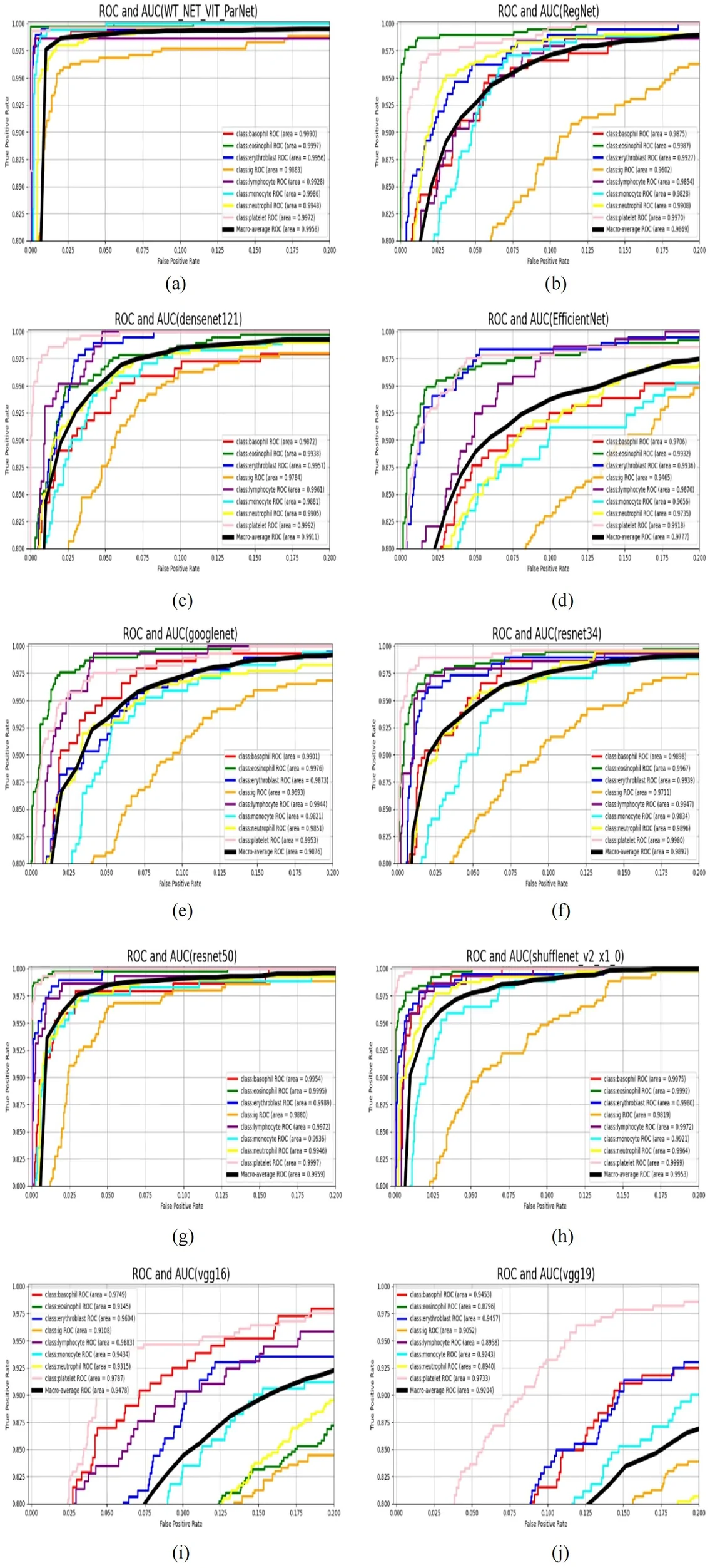
Comparison of ROC Curves for Public Datasets **(a)**–**(j)** are the ROC curves for the PV-WTConv, RegNet, DenseNet121, EfficientNet, GoogLeNet, ResNet34, ResNet50, ShuffleNet_v2_x1_0, VGG16 and VGG19 networks, respectively.

### 5.3 Visualisation and class analysis

To combine qualitative observations with quantitative results, Figure 13 shows the results of overlaying heatmaps from different models on selected examples of the six cell types in the AWBC-LZWH dataset. Each model exhibits a distinct spatial distribution of responses, which can be used to aid in observing their response regions within these examples. These visualisations constitute a qualitative description; they cannot, on their own, prove that feature localisation is more accurate or that a particular model is superior, and should be interpreted in conjunction with the classification metrics of the test set and the results of category-level evaluations.

**Figure 13:**
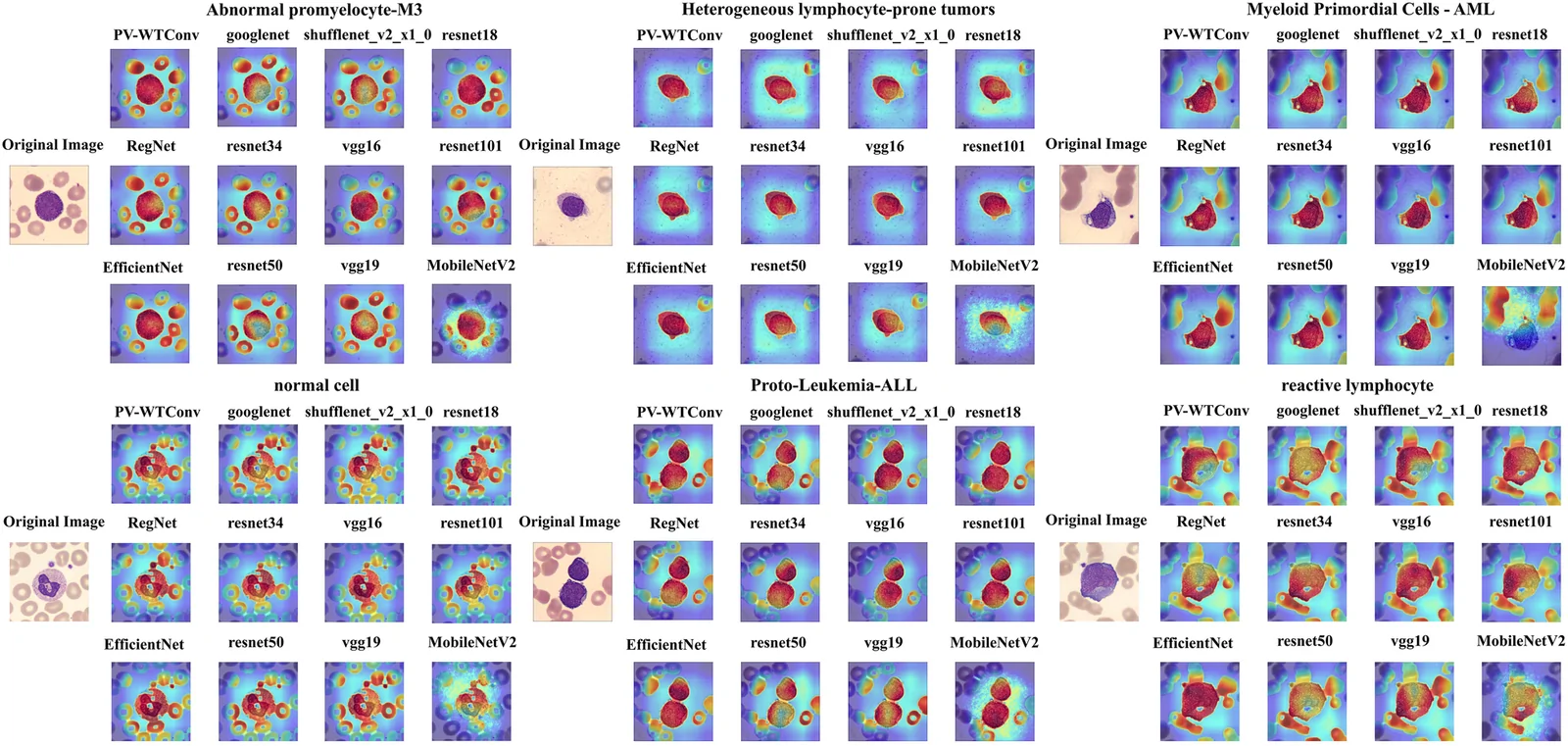
Overlaid visualisation of heatmaps from different models on images of six white blood cell types in the AWBC-LZWH dataset.

To further evaluate the model’s ability to recognise different categories, Tables 8, 9 report the Precision, Recall and F1-score for each category of PV-WTConv across the two datasets, respectively. All three metrics for each category in the AWBC-LZWH dataset were at least 0.92, whilst those in the PBC_dataset reached values above 0.927 for each category, providing a class-wise description of the model’s performance under the evaluated conditions.

**Table 8:** Experimental results of the PV-WTConv network for the six categories on the AWBC-LZWH dataset.

Class	Precision	F1	Recall
Abnormal promyelocytes in acute promyelocytic leukemia (APL; FAB M3)	0.92305	0.93634	0.95000
Abnormal lymphocytes (suspected neoplastic)	0.98880	0.97418	0.96000
Myeloblasts (AML)	0.92221	0.92114	0.92000
Lymphoblasts (ALL)	0.92000	0.92000	0.92000
Normal leukocytes	0.97917	0.95918	0.94000
Reactive lymphocytes	0.94231	0.96078	0.98000

**Table 9:** Experimental results of the PV-WTConv network across eight categories on the PBC_dataset.

Class	Precision	F1	Recall
Basophil	0.96478	0.96738	0.97000
Eosinophil	1.00000	1.00000	1.00000
Erythroblast	0.95971	0.97462	0.99000
Ig	0.92759	0.92880	0.93000
Lymphocyte	0.95240	0.96112	0.97000
Monocyte	0.97074	0.96534	0.96000
Neutrophil	0.97327	0.96149	0.95000
Platelet	0.99336	0.99168	0.99000

### 5.4 Limitations of the study

Although PV-WTConv achieved good classification performance, this study still has certain limitations. The AWBC-LZWH dataset originates from a single clinical centre; differences may exist between different medical institutions in terms of microscopic imaging equipment, staining conditions, sample preparation processes and patient populations. Therefore, further multi-centre, cross-device and cross-domain validation is required. In this study, patient-level grouping was employed to ensure that there was no overlap in patient identities between the training, validation and test sets of the AWBC-LZWH dataset, thereby preventing the leakage of patient-level information caused by multiple cell images from the same patient being included in different data subsets simultaneously. However, as the AWBC-LZWH dataset originates from a single clinical centre, differences may exist between different healthcare institutions in terms of patient populations, sample preparation processes, staining conditions and microscopy equipment. Whilst patient-independent testing can evaluate the model’s classification performance on images from patients at the same centre who were not involved in training or validation, it cannot replace external validation based on independent medical centres and independent patient cohorts. Consequently, multi-centre, cross-device and cross-domain evaluations are still required to further validate the model’s generalisation ability in different clinical settings. This study primarily validated the algorithm using existing image datasets and has not yet undergone prospective clinical validation within real-world clinical workflows; consequently, the current results cannot directly demonstrate the model’s clinical efficacy. Compared with lightweight networks, the introduction of ViT and ParNetAttention increases the number of model parameters and entails some computational overhead. Future research could explore model pruning, knowledge distillation or lightweight attention structures to improve the model’s deployment efficiency. Furthermore, this study employs a uniform set of hyperparameters to compare different networks, thereby ensuring consistent training and evaluation conditions. However, the same learning rate, batch size, number of training iterations and other hyperparameters may not be optimal for all architectures; different networks may have varying requirements in terms of optimal configurations and training budgets. Consequently, the results presented here reflect the relative performance of each model under the reported uniform settings for training from scratch and do not represent the best performance that each architecture could achieve following individual, thorough hyperparameter tuning. Future research could optimise the training configurations of each architecture separately, based on the validation set and within a comparable hyperparameter search budget, to further assess the robustness of the comparative results. Regarding specialised white blood cell classification models proposed in recent years, direct comparisons under a unified dataset and standardised experimental protocols should be conducted, provided that their source code is available and consistency in data partitioning, image pre-processing and evaluation metrics can be ensured. It should be further noted that, as the PBC_dataset and AWBC-LZWH employ different cell classification schemes, neither the independent eight-class classification experiments on the PBC_dataset nor the cross-dataset transfer experiments from the PBC_dataset to AWBC-LZWH should be interpreted as external validation of the original AWBC-LZWH six-class classification model. These experiments serve, respectively, to evaluate the suitability of the network architecture for another classification task and the cross-task transferability of the learned features. Rigorous external validation of the original six-class classifier still requires the use of an external cohort that is independent of the source, independent of patients or samples, and comprises the same six target categories; the six-class classifier developed using AWBC-LZWH must be evaluated directly without retraining the model using this external cohort. Such validation will form an important component of subsequent multicentre studies. Future efforts will focus on multi-centre external validation and clinical validation in real-world clinical settings, whilst further optimising the model’s computational efficiency to assess its potential value as a clinical aid. Although PV-WTConv achieved satisfactory classification results on the AWBC-LZWH and PBC_dataset, this study primarily constitutes an evaluation of offline algorithms based on existing microscopic image data and has not yet undergone prospective, multicentre clinical validation. The AWBC-LZWH dataset originates from a single medical centre; differences between institutions in terms of patient populations, microscopic imaging equipment, staining conditions and sample preparation procedures may affect the model’s performance in other clinical settings. Furthermore, whilst supplementary training and testing conducted on the PBC_dataset provide algorithm evaluation results under a different data distribution, they cannot substitute for prospective, multicentre external validation of the same model. Consequently, the image-level classification metrics reported in this paper are insufficient to demonstrate the model’s diagnostic efficacy at the patient level, nor can they be used directly to judge its effectiveness and practical clinical benefits within real-world clinical workflows. At this stage, PV-WTConv should be regarded as a white blood cell image classification method with value for further research and validation; its value as a clinical aid remains to be assessed, and the current experimental results should not be interpreted as indicating that the model is ready for clinical diagnostic application. In light of the aforementioned limitations, the research team is currently collecting prospective data and datasets from other medical centres, and will continue to advance this work, prioritising prospective and multicentre validation in subsequent studies. Once data of sufficient scale and quality to meet research requirements has been obtained, the plan is to conduct validation using samples independent of the model development data, focusing on assessing the model’s classification stability and generalisation ability across different centres, patient populations, imaging equipment and staining conditions, whilst further examining its applicability and misclassification rates within real-world clinical workflows. Data collection is currently underway; the relevant validation remains part of future research, and its results have not been included in this manuscript nor are they used as a basis for assessing current clinical utility. We will conduct a further objective assessment of the clinical utility and scope of application of this method based on the results of subsequent validation studies.

## Data Availability

The original contributions presented in the study are included in the article/Supplementary Material, further inquiries can be directed to the corresponding author.
